# Supplementary material for: A temperature sensor with a wide spectral range based on a dual-emissive TADF dendrimer system
Source: Nat Commun. 2024 Aug 28;15:7439. doi: 10.1038/s41467-024-51231-x (PMC11358277; doi:10.1038/s41467-024-51231-x)
Supplement: Supplementary file 1 — Supplementary Information [file 41467_2024_51231_MOESM1_ESM.pdf]

Supplementary Information

A Temperature Sensor with a Wide Spectral Range Based on a Dual-emissive TADF Dendrimer System

C. Si et al.

Contents

Supplementary Methods **3**

Supplementary Notes **27**

DFT Calculations 27

Photophysical Properties 30

OLED Characterisation 56

Supplementary References **61**

Supplementary Methods

General Synthetic Procedures

The following compounds were synthesised according to the literature: N^3,N^3,N^6,N^6-tetraphenyl-9*H*-arbazole-3,6-diamine (GCz),[1] and 3,6-dibromo-9,10-phenanthrenequinone.[2] All commercially available chemicals and reagent grade solvents were used as received. Air-sensitive reactions were performed under a nitrogen atmosphere using Schlenk techniques. Flash column chromatography was carried out using silica gel (Silia-P from Silicycle, 60 Å, 40-63 μm). Analytical thin-layer-chromatography (TLC) was performed with silica plates with aluminum backings (250 μm with F-254 indicator). TLC visualization was accomplished by 254/365 nm UV lamp. ^{1}H and ^{13}C NMR spectra were recorded on a Bruker Avance spectrometer (400 or 500 MHz for ^{1}H, 101 or 126 MHz for ^{13}C). The following abbreviations have been used for multiplicity assignments: "s" for singlet, "d" for doublet, and "dd" for doublet of doublets. Deuterated chloroform ($CDCl_3$) and THF-d_8 were used as the solvents of record. ^{1}H NMR and ^{13}C NMR spectra were referenced to the solvent peak. Melting points were measured using open-ended capillaries on an Electrothermal melting point apparatus IA9200 and are uncorrected. HPLC analysis was conducted on a Shimadzu LC-40 HPLC system. HPLC traces were performed using a Shim-pack GIST 3μm C18 reverse phase analytical column. High-resolution mass spectrometry (HRMS) was performed at the University of Edinburgh. Elemental analyses (EA) were performed by the School of Geosciences at the University of Edinburgh.

Literature Study

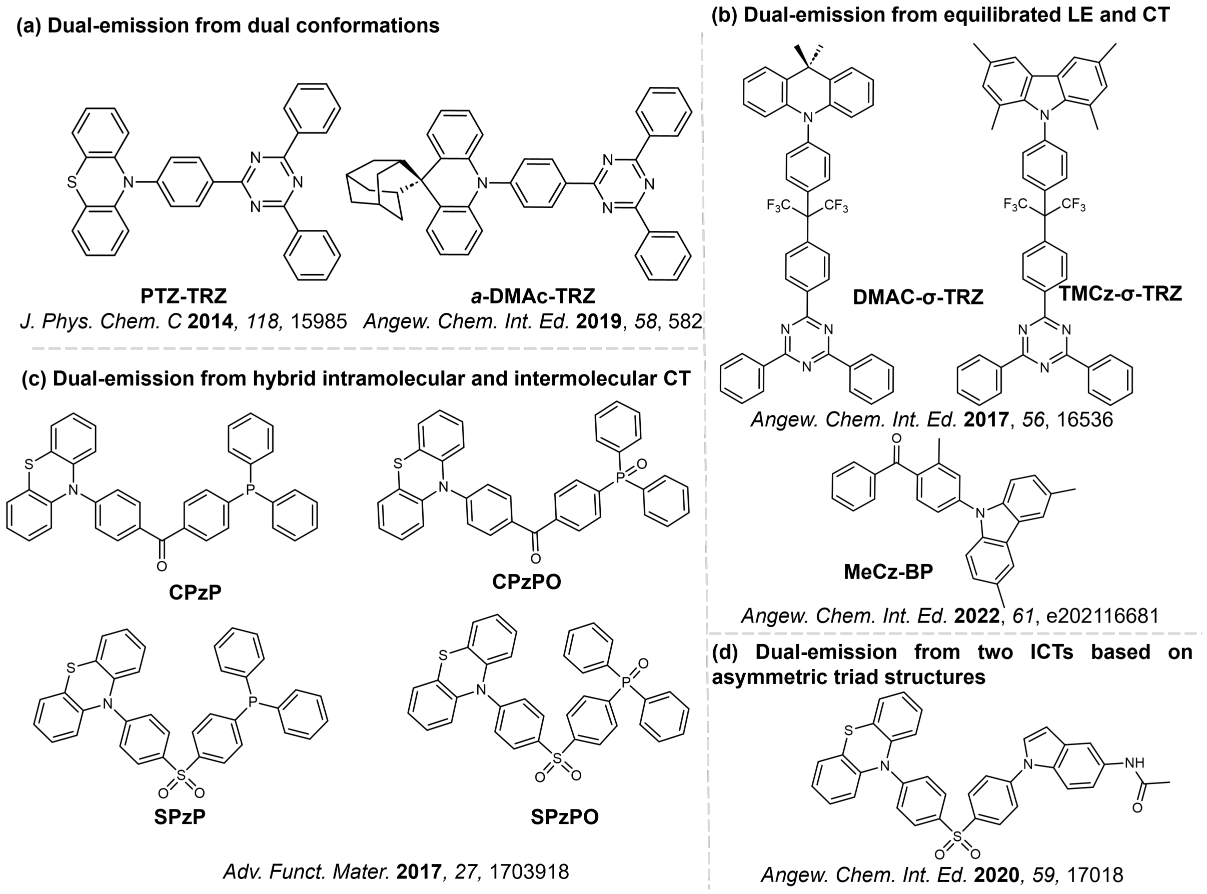

Supplementary Fig. 1. Examples of Dual dual emission from (a) dual conformations, (b) equilibrated LE and CT states, (c) "hybrid intramolecular and intermolecular CT" states and (d) two ICT states based on asymmetric triad structures.

Chemical Synthesis

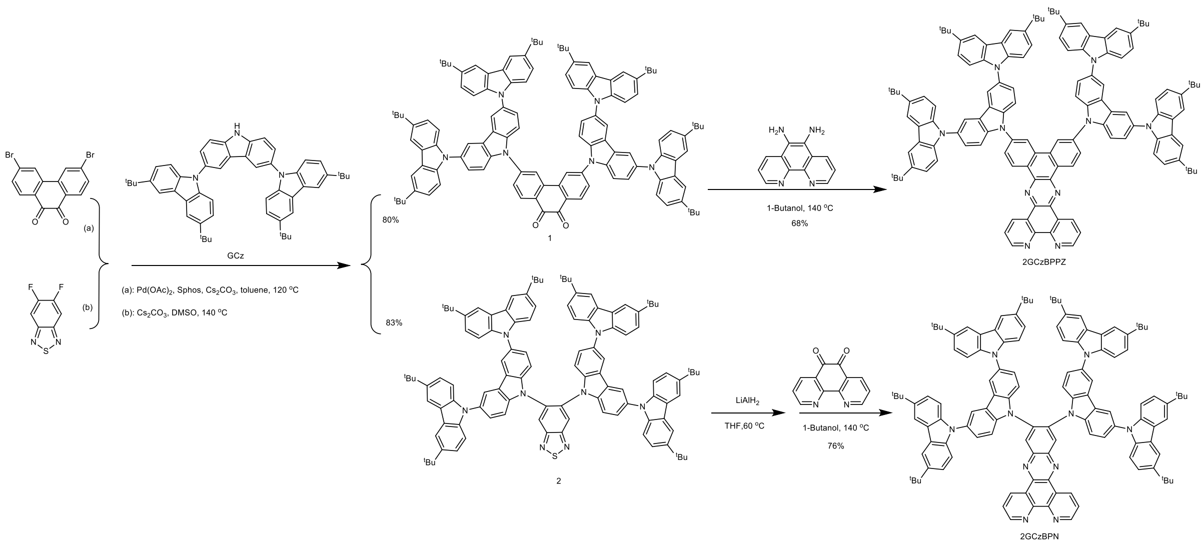

Supplementary Fig. 2. Synthetic Routes for 2GCzBPPZ and 2GCzBPN.

Synthesis of 3,6-bis(3,3'',6,6''-tetra-*tert*-butyl-9'*H*-[9,3':6',9''-tercarbazol]-9'-yl)phenanthrene-9,10-dione (1):

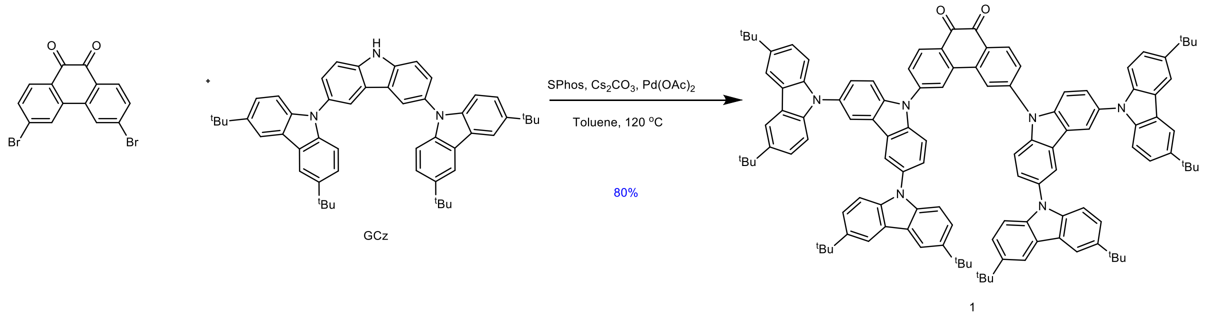

Under a nitrogen atmosphere, to a 100 mL Schlenk flask were added 3,6-dibromo-9,10-phenanthrenequinone (0.5 g, 1.4 mmol, 1.0 equiv.), **GCz** (2.2 g, 3.0 mmol, 2.2 equiv.), $Pd(OAc)_2$ (0.015 g, 0.07 mmol, 0.05 equiv.), 2-dicyclohexylphosphino-2′,6′-dimethoxybiphenyl (SPhos) (0.084 g, 0.2 mmol, 0.15 equiv.), Cs_2CO_3 (2.7 g, 8.2 mmol, 6.0 equiv.) and toluene (50 mL) and the reaction was stirred at 110 °C for 24 h. After being cooled to room temperature, the reaction mixture was added to aqueous NH_4Cl (30 mL) and was extracted with DCM (3×50 mL). The organic layer was dried over anhydrous Na_2SO_4, filtered, and concentrated under reduced pressure to afford around 2.0 g of the crude product as a dark solid. The crude product was purified by column chromatography on silica gel (25% DCM/hexane) to afford **1** as a dark red solid (Yield =1.80 g).

Yield: 80%. R_f = 0.2 (25% DCM/hexane). Mp = 341-343 °C. ^{1}H NMR (500 MHz, $CDCl_3$) δ 8.68 (d, J = 8.3 Hz, 2H), 8.46 (s, 2H), 8.26 (s, 4H), 8.13 (s, 8H), 7.99 (d, J = 9.3 Hz, 2H), 7.76 (d, J = 8.7 Hz, 4H), 7.65 (d, J = 8.7 Hz, 4H), 7.41 (d, J = 8.7 Hz, 8H), 7.29 (d, J = 8.6 Hz, 8H), 1.43 (s, 72H). ^{13}C NMR (126 MHz, $CDCl_3$): 142.83, 139.96, 139.35, 132.25, 126.53, 124.97, 123.65, 123.25, 119.75, 116.30, 111.11, 108.93, 77.27, 77.02, 76.77, 34.72, 32.01. HR-MS $[M+H]^+$ Calculated: ($C_{118}H_{114}N_6O_2$) 1646.9031; Found: 1646.9016.

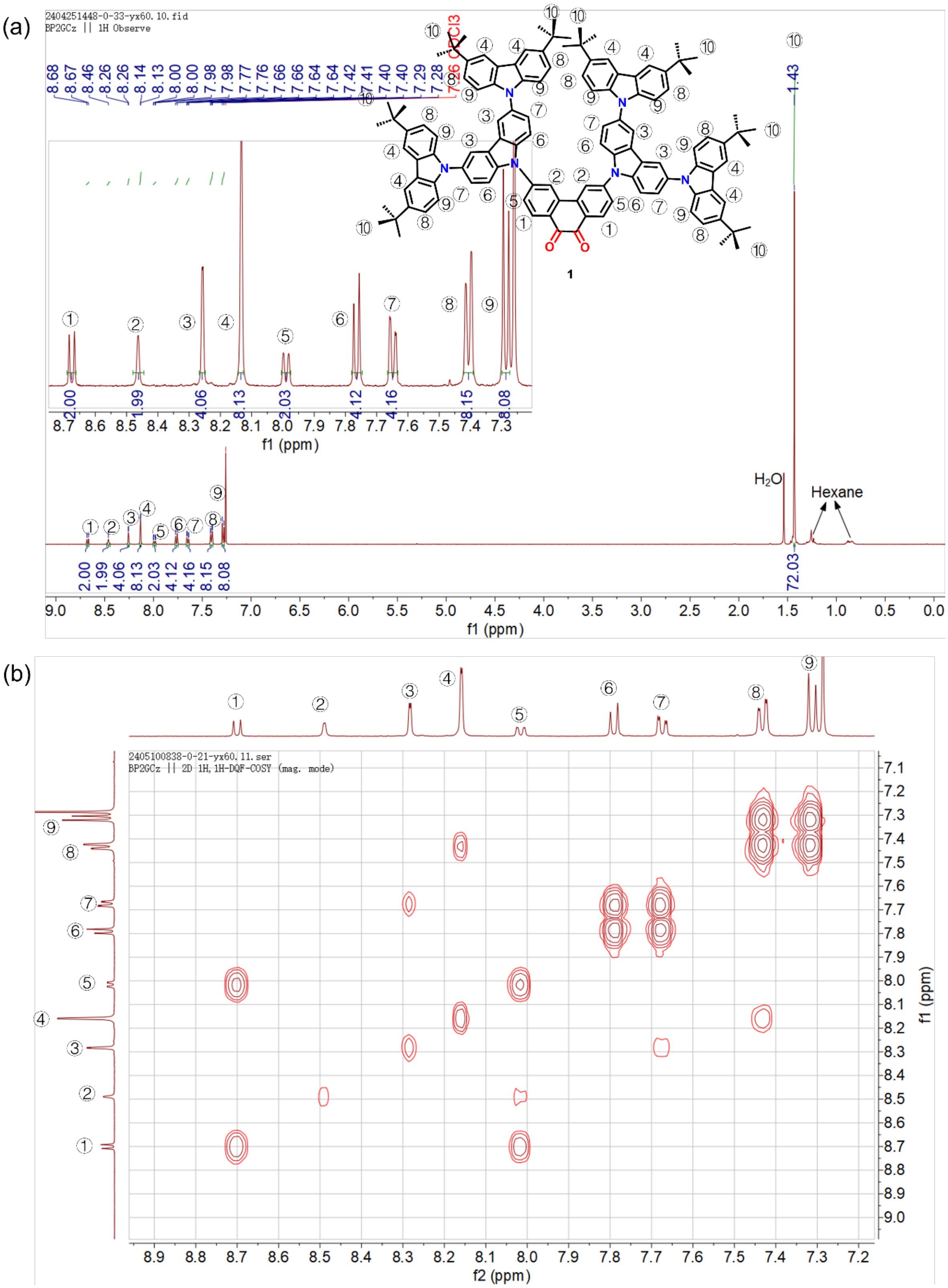

Supplementary Fig. 3. (a) 1D ^{1}H NMR and (b) ^{1}H-^{1}H COSY NMR spectra of **1** in $CDCl_3$.

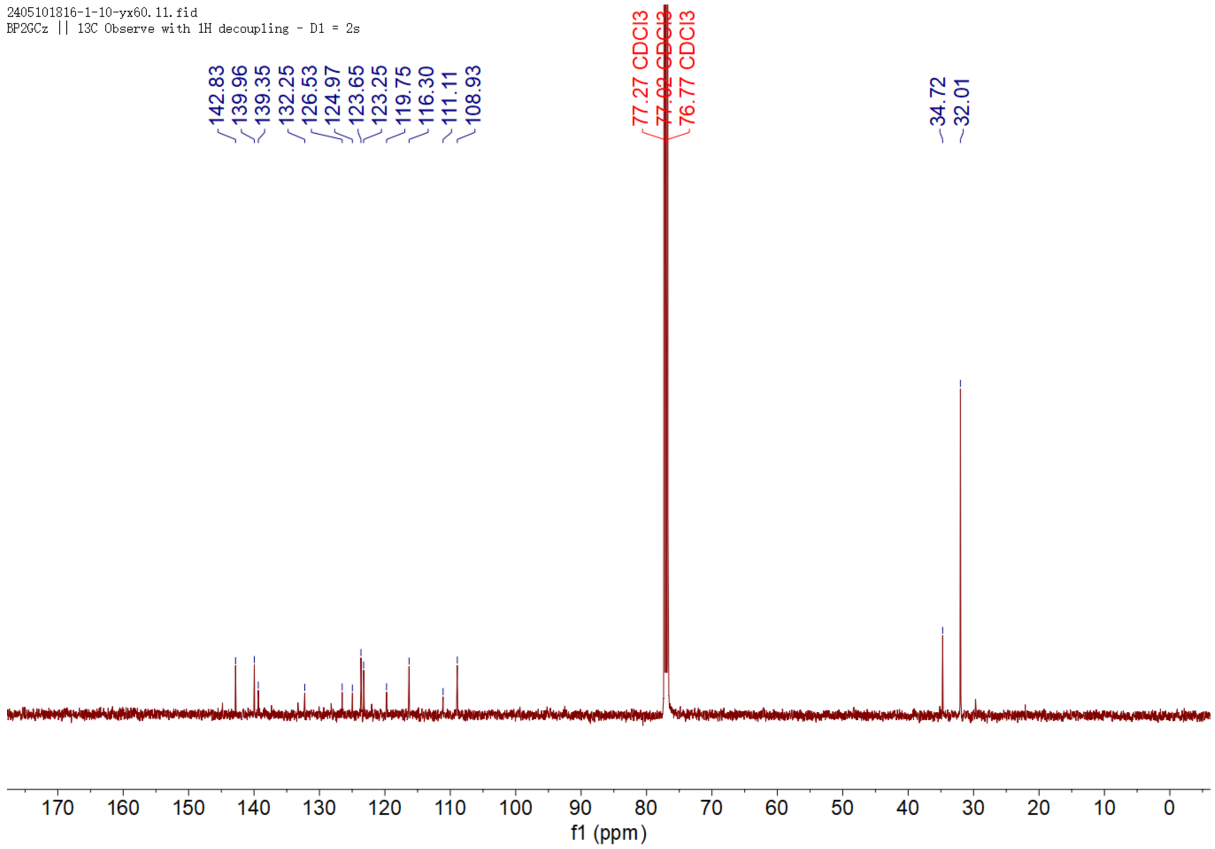

Supplementary Fig. 4. ^{13}C NMR spectra of **1** in $CDCl_3$.

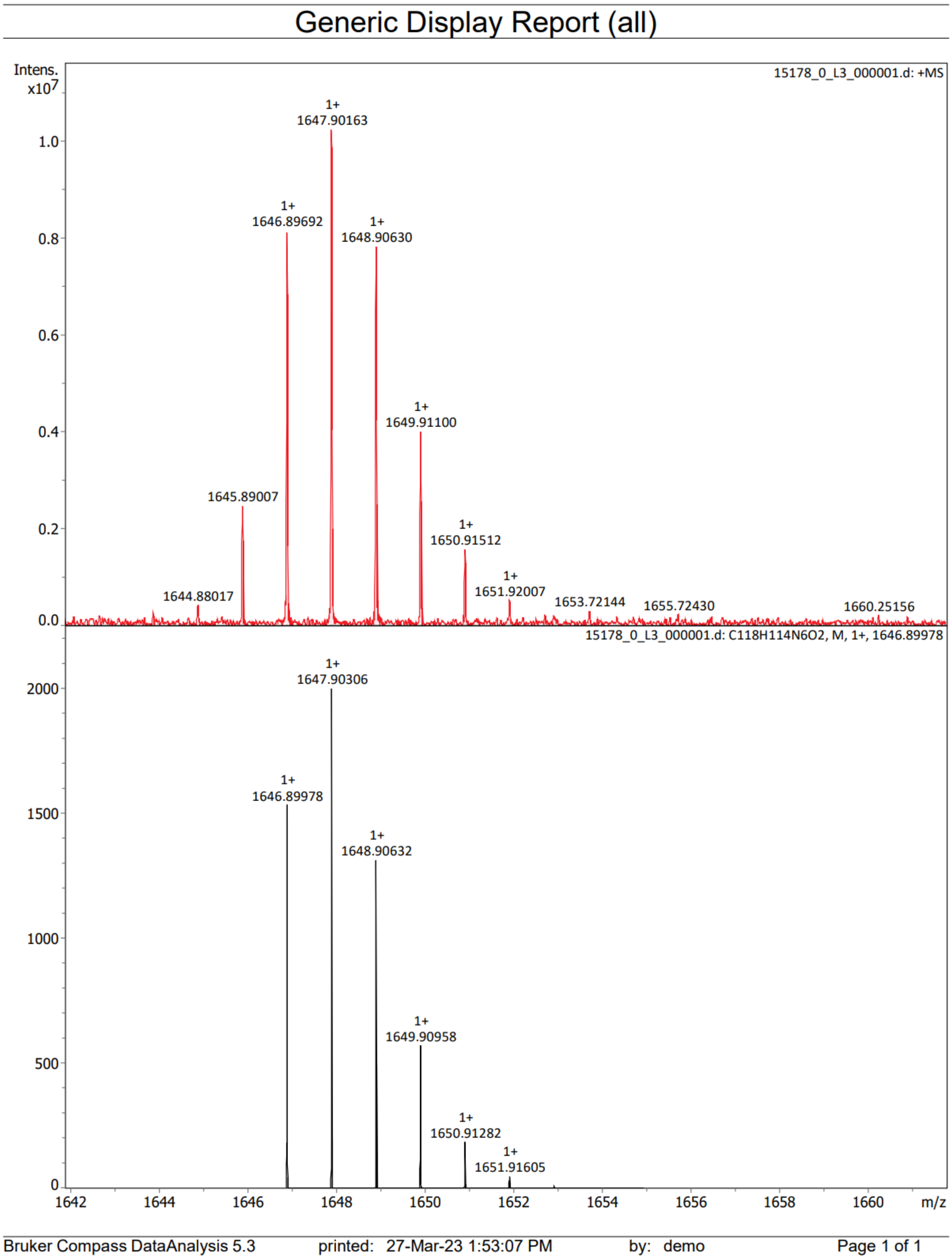

Supplementary Fig. 5. HRMS of **1**.

Synthesis of 5,6-bis(3,3'',6,6''-tetra-*tert*-butyl-9'*H*-[9,3':6',9''-tercarbazol]-9'-yl)benzo[*c*][1,2,5]thiadiazole (2):

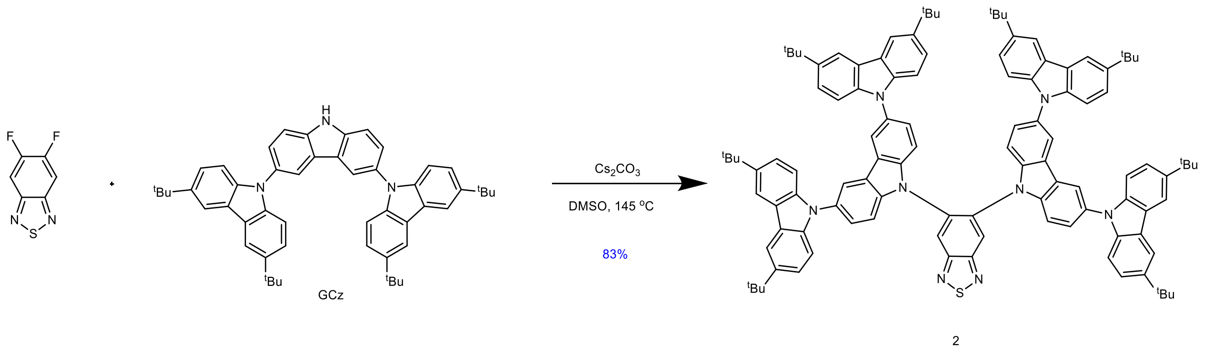

To a flask containing 10 mL of DMSO were added 5,6-difluorobenzo[*c*][1,2,5]thiadiazole (0.4 g, 2.32 mmol, 1.0 equiv.), GCz (3.52 g, 4.88 mmol, 2.1 equiv.) and Cs_2CO_3 (3.79 g, 11.62 mmol, 5.0 equiv.) and the reaction was heated to 145 °C and stirred under a nitrogen atmosphere for 16 h. After cooling to room temperature, the solution was poured into water (50 mL) and extracted with DCM (3×100 mL). The organic layer was dried over Na_2SO_4, filtered, and concentrated under reduced pressure. The residue (around 3.5 g of a dark yellow solid) was purified by column chromatography with (20% DCM/hexane) to afford the compound **2** as a light-yellow solid (Yield: 3.05 g).

Yield: 83%. R_f = 0.4 (20% DCM/Hexane). Mp > 400 °C. ^{1}H NMR (500 MHz, THF-d_8) δ 9.04 (s, 2H), 8.17 (s, 4H), 8.11 (s, 8H), 7.53 (d, J = 8.6 Hz, 4H), 7.29 (d, J = 8.6 Hz, 4H), 7.08 (s, 16H), 1.32 (s, 72H). ^{13}C NMR (126 MHz, THF-d_8): δ 152.77, 140.07, 137.98, 137.76, 129.55, 122.86, 122.57, 121.79, 121.50, 121.23, 116.99, 113.96, 109.67, 106.89, 32.42, 29.58. HR-MS $[M+H]^+$ Calculated: ($C_{110}H_{110}N_8S$) 1574.8601; Found: 1574.8574.

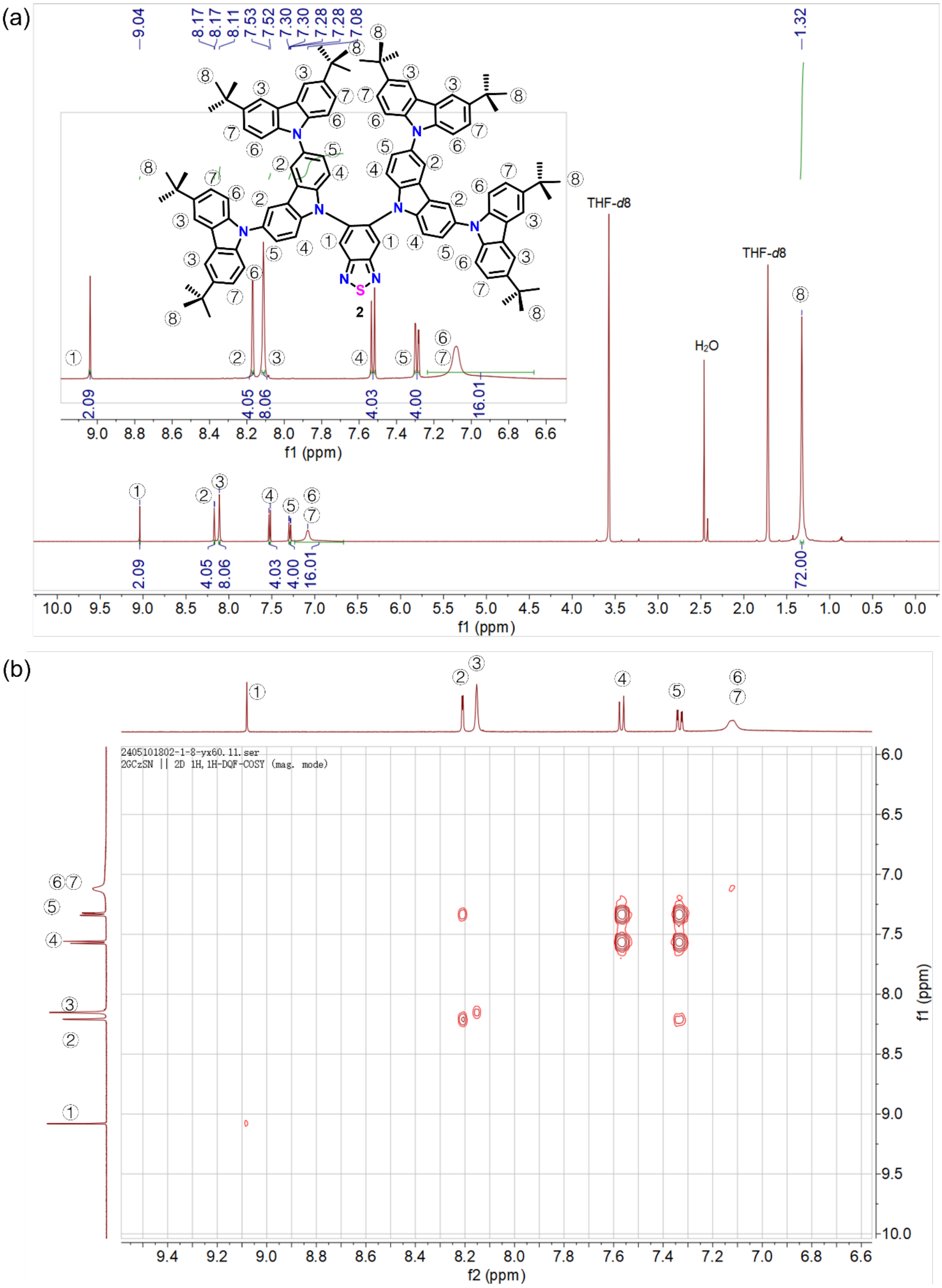

Supplementary Fig. 6. (a) 1D ^{1}H NMR and (b) ^{1}H-^{1}H COSY NMR spectra of **2** in THF-d_8.

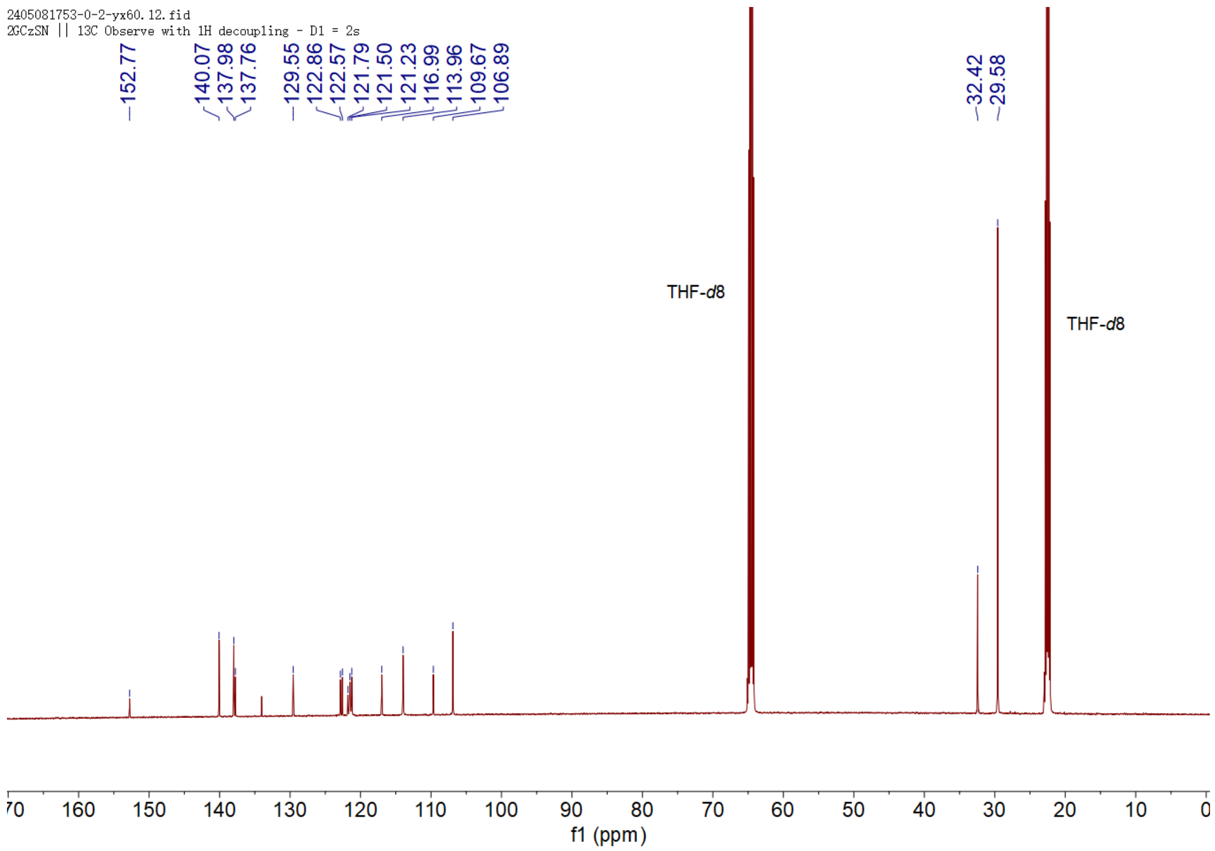

Supplementary Fig. 7.^{13}C NMR spectra of **2** in THF-d_8.

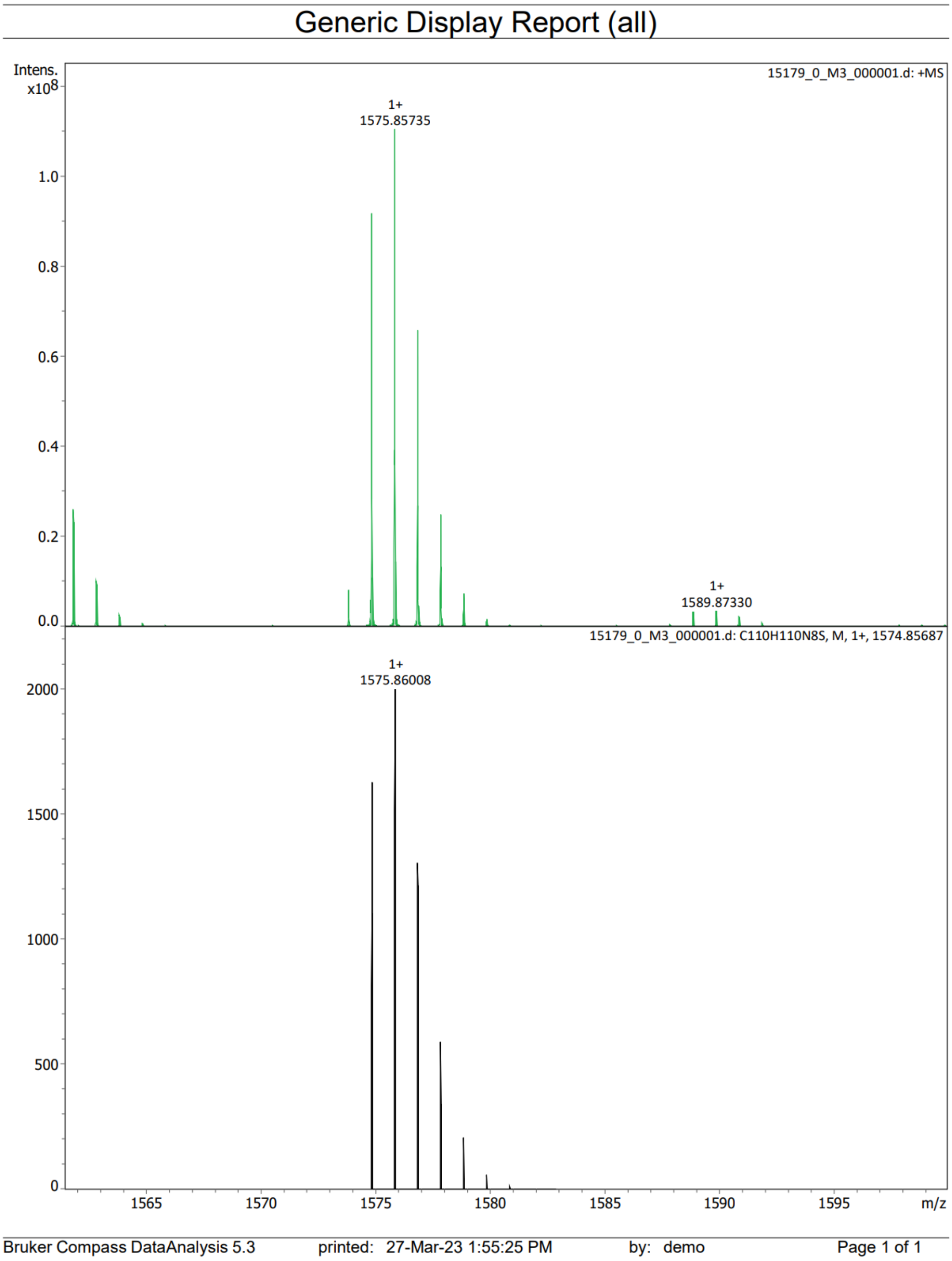

Supplementary Fig. 8. HRMS of **2**.

Synthesis of 12,15-bis(3,3'',6,6''-tetra-*tert*-butyl-9'*H*-[9,3':6',9''-tercarbazol]-9'-yl)dibenzo[*a,c*]dipyrido[3,2-*h*:2',3'-*j*]phenazine (2GCzBPPZ):

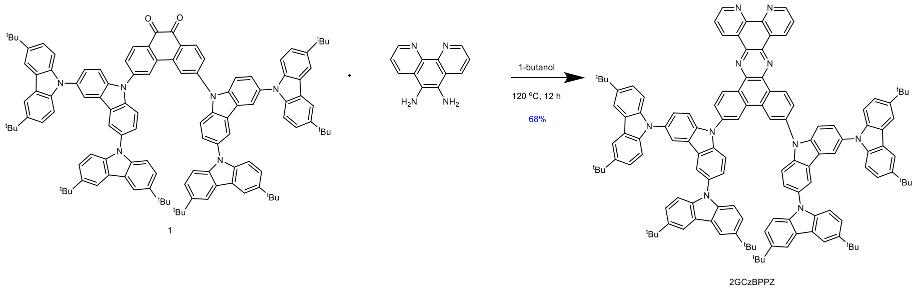

To a flask containing 10 mL of 1-butanol were added **1** (0.8 g, 0.48 mmol, 1.0 equiv.) and 1,10-phenanthroline-5,6-diamine (0.1 g, 0.48 mmol, 1.0 equiv.) and the reaction was heated to 120 °C for 12h under a nitrogen atmosphere. After cooling to room temperature, the solution was poured into water (20 mL) and extracted with DCM (3×50 mL). The organic layer was dried over Na_2SO_4, filtered, and concentrated under reduced pressure. The residue (around 0.7 g of a yellow solid) was purified by column chromatography with (20% DCM/hexane) to afford the compound 2GCzBPPZ as a yellow solid (0.60 g).

Yield: 68%. R_f = 0.3 (20% DCM/hexane). Mp >400 °C. ^{1}H NMR (500 MHz, $CDCl_3$) δ10.05 (d, *J* = 8.5 Hz, 2H), 9.97 (d, *J* = 8.2 Hz, 2H), 9.42 (d, *J* = 4.5 Hz, 2H), 9.07 (s, 2H), 8.32 (d, J=1.8 Hz, 2H), 8.31 (s, 4H), 8.15 (s, 8H), 7.98 (dd, *J* = 8.1, 4.4 Hz, 2H), 7.82 (d, *J* = 8.7 Hz, 4H), 7.66 (d, *J* = 8.7 Hz, 4H), 7.43 (d, *J* = 8.8 Hz, 8H), 7.34 (d, *J* = 8.6 Hz, 8H), 1.44 (s, 72H). ^{13}C NMR (126 MHz, $CDCl_3$): δ 152.52, 142.72, 140.96, 140.39, 140.14, 139.68, 133.93, 133.16, 131.66, 129.88, 128.79, 127.70, 127.54, 126.40, 124.48, 123.62, 123.22, 121.73, 119.71, 116.26, 111.02, 109.04, 77.26, 77.01, 76.76, 34.72, 32.03. HR-MS $[M+H]^+$ Calculated: ($C_{130}H_{120}N_{10}$) 1821.9802; Found: 1821.9893. Anal. Calcd. for $C_{130}H_{120}N_{10}$: C, 85.68%; H, 6.64%; N, 7.69%. Found: C, 85.77%; H, 6.84%; N, 7.68%. HPLC analysis: 98.6% pure on HPLC analysis, retention time 10.190 minutes in mixture of 85% Acetonitrile and 15% Water.

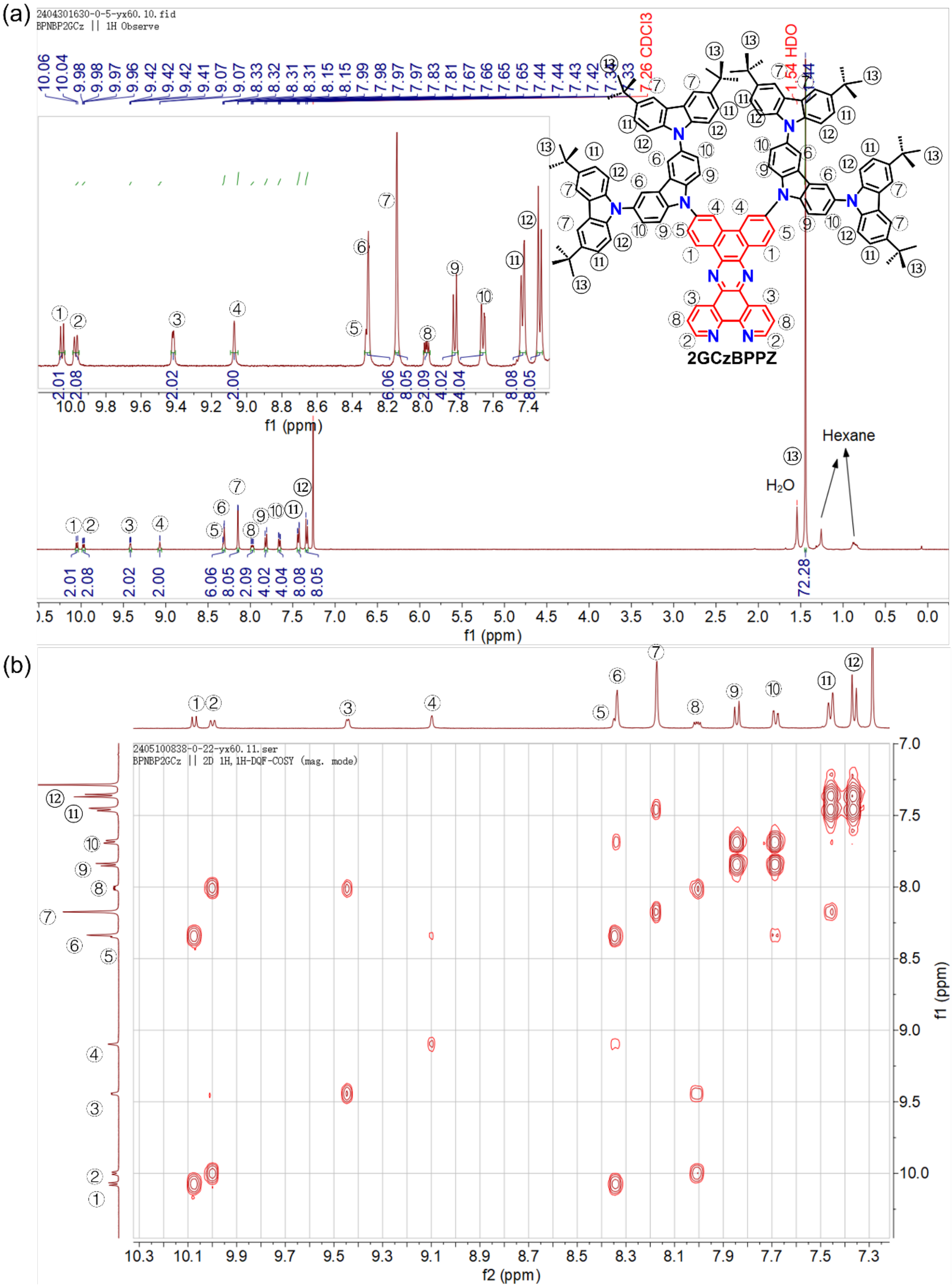

Supplementary Fig. 9. (a) 1D ^{1}H NMR and (b) ^{1}H-^{1}H COSY NMR spectra of 2GCzBPPZ in $CDCl_3$.

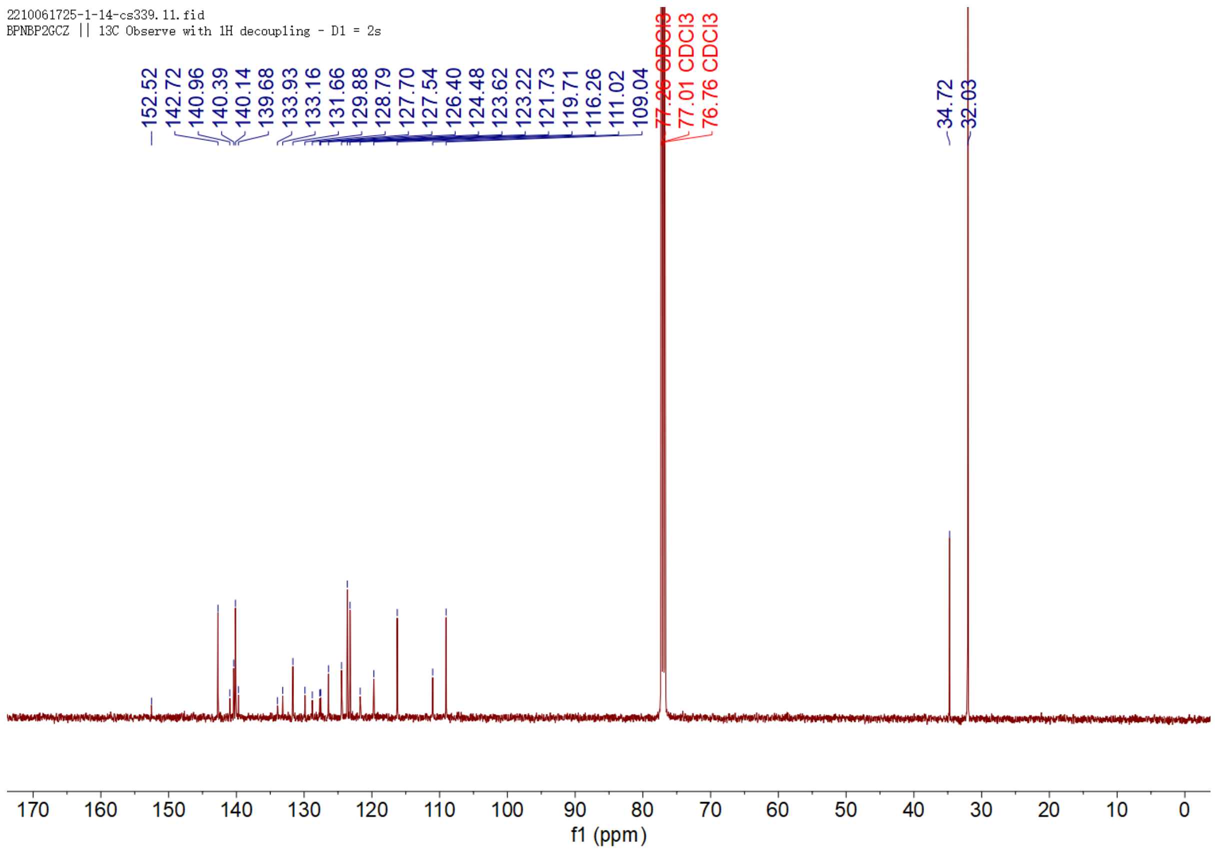

Supplementary Fig. 10. ^{13}C NMR spectra of 2GCzBPPZ in $CDCl_3$.

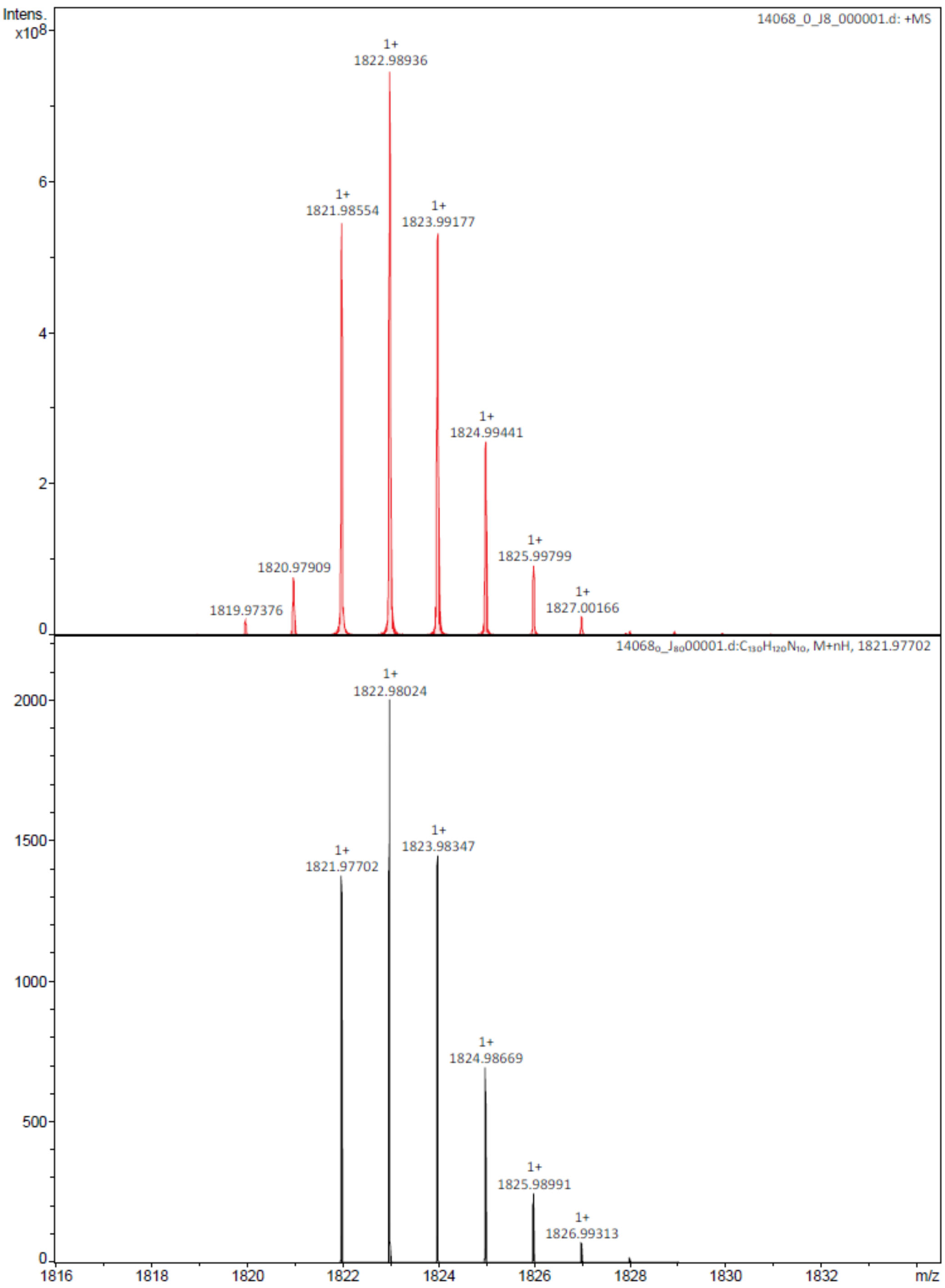

Supplementary Fig. 11. HRMS of 2GCzBPPZ.

Elemental Analysis Service Request Form

Researcher name Changfeng Si

Researcher email cs339@st-andrews.ac.uk

NOTE: Please submit ca. 10 mg of sample

**Sample reference number**	CS289-BPNBP2GCz
**Name of Compound**	BPNBP2GCz
**Molecular formula**	C130H120N10
**Stability**	
**Hazards**	
**Other Remarks**	

Analysis type:

Single ☐ **Duplicate** ■ **Triplicate** ☐

Analysis Result:

Element	Expected %	Found (1)	Found (2)	Found (3)
Nitrogen	7.69	7.70	7.78	
Carbon	85.68	85.77	86.20	
Hydrogen	6.64	6.84	6.88	

Authorising Signature:

**Date completed**	28.11.22
**Signature**	J-P C
**comments**	Revised + signed 09.05.24 after an error was found w/ the molecular formula. JPC 09.05.24

Supplementary Fig. 12. Elemental analysis of 2GCzBPPZ.

HPLC Trace Report31Mar2023

<Sample Information>

Sample Name : BPNBP2GCZ
Sample ID :
Method Filename : 85% Acetonitrile 15 Water 20 mins.lcm
Batch Filename : BPNBP2GC.lcb
Vial # : 1-23
Sample Type : Unknown
Injection Volume : 20 uL
Date Acquired : 31/03/2023 21:37:26
Acquired by : System Administrator
Date Processed : 31/03/2023 21:57:28
Processed by : System Administrator

<Chromatogram>

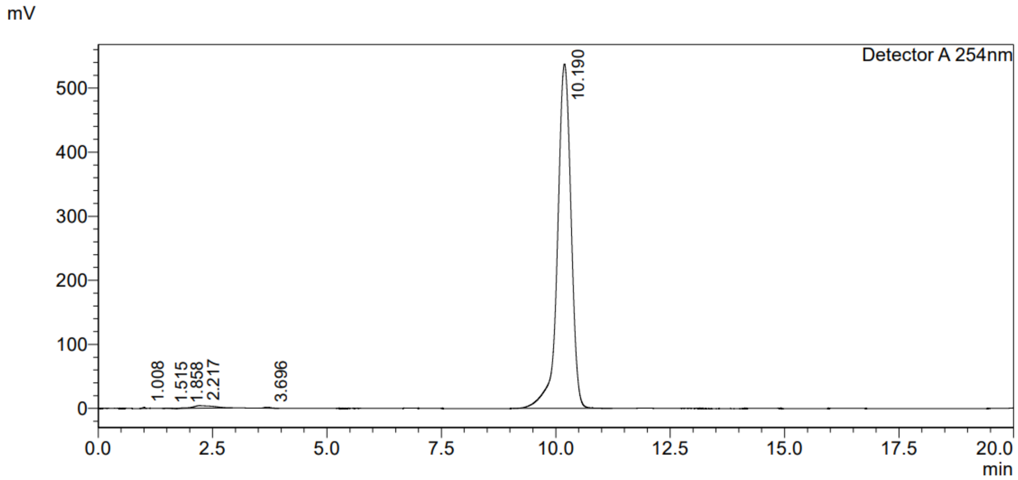

<Peak Table>

Detector A 254nm

Peak#	Ret. Time	Area	Height	Area%	Area/Height	Width at 5% Height
1	1.008	5216	1521	0.046	3.429	0.119
2	1.515	4148	344	0.037	12.050	0.271
3	1.858	7836	870	0.070	9.006	--
4	2.217	124941	4070	1.110	30.699	--
5	3.696	14751	1534	0.131	9.616	0.321
6	10.190	11099732	537490	98.606	20.651	0.767
Total		11256625	545830	100.000		

Supplementary Fig. 13. HPLC trace of 2GCzBPPZ.

Synthesis of 11,12-bis(3,3'',6,6''-tetra-*tert*-butyl-9'*H*-[9,3':6',9''-tercarbazol]-9'-yl)dipyrido[3,2-*a*:2',3'-*c*]phenazine (2GCzBPN):

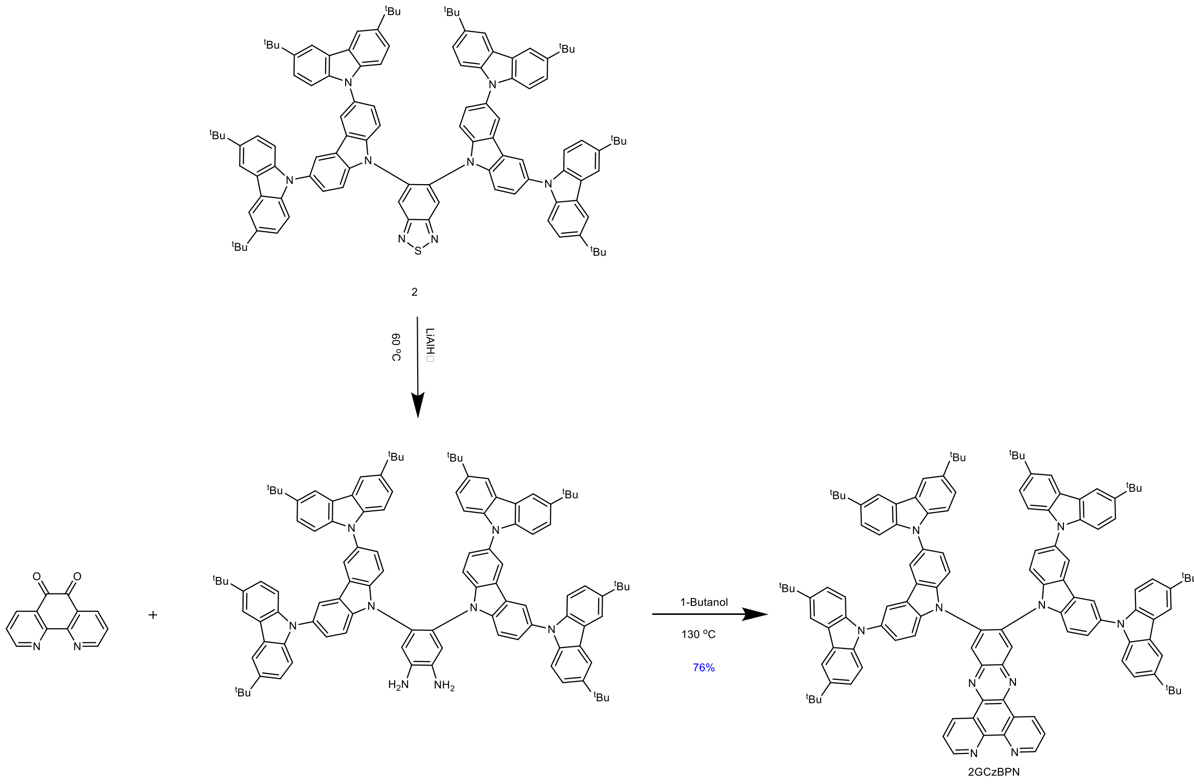

To a solution of **2** (1.0 g, 0.63 mmol, 1.0 equiv.) in dry THF (40 mL) were added lithium aluminum hydride (0.19g, 5.08 mmol 8.0 equiv.) under a continuous N_2 flow. The resulting mixture was stirred at 60 °C under N_2 atmosphere for 2 h. After cooling to 0 °C, the reaction mixture was quenched with water (2 mL) and then 2 M NaOH(aq) (2 mL) was added. The solution was filtered through a pad of Celite, which was subsequently rinsed with ethyl acetate (3×50 mL), and the filtrate was then extracted with ethyl acetate (3×50 mL). The organic layer was collected and dried over $MgSO_4$(s). After filtration and removal of the solvent under reduced pressure, compound **3** was used directly for the next step without further purification. Compound **3** and 1,10-phenanthroline-5,6-dione (0.13 g, 0.63 mmol, 1.0 equiv.) were added into 40 mL of 1-butanol and then heated to reflux for 12h under a nitrogen atmosphere. After cooling to room temperature, the solution was poured into water and extracted with DCM (3 × 100 mL). The organic layer was dried over Na_2SO_4, filtered, and concentrated under reduced pressure. The residue (around 1.0 g of an orange solid) was purified by column chromatography with (25% DCM/hexane) to afford the compound 2GCzBPN as a yellow solid (0.83 g).

Yield: 76%. R_f = 0.3 (25% DCM/Hexane). Mp > 400 °C. ^{1}H NMR (500 MHz, $CDCl_3$) δ 9.87 (d, J = 8.1 Hz, 2H), 9.46 (d, J = 3.6 Hz, 2H), 9.23 (s, 2H), 8.11 (s, 8H), 8.05 (s, 4H), 7.98 (dd,

J = 8.1, 4.4 Hz, 2H), 7.49 (d, *J* = 8.6 Hz, 4H), 7.33 (d, *J* = 8.7 Hz, 4H), 7.09 (s, 16H), 1.37 (s, 72H). ^{13}C NMR (126 MHz, $CDCl_3$): δ 153.43, 148.91, 142.90, 142.62, 142.22, 139.77, 139.29, 136.28, 134.24, 131.85, 131.53, 127.24, 125.34, 124.61, 124.52, 123.74, 123.13, 118.97, 116.19, 111.17, 108.73, 77.36, 77.11, 76.86, 34.68, 32.05. HR-MS $[M+H]^+$ Calculated: ($C_{122}H_{116}N_{10}$) 1721.9489; Found: 1721.9672. Anal. Calcd. for $C_{122}H_{116}N_{10}$: C, 85.08%; H, 6.79%; N, 8.13%. Found: C, 85.23%; H, 6.96%; N, 8.29%. HPLC analysis: 99.2% pure on HPLC analysis, retention time 6.448 minutes in mixture of 90% Acetonitrile and 10% Water.

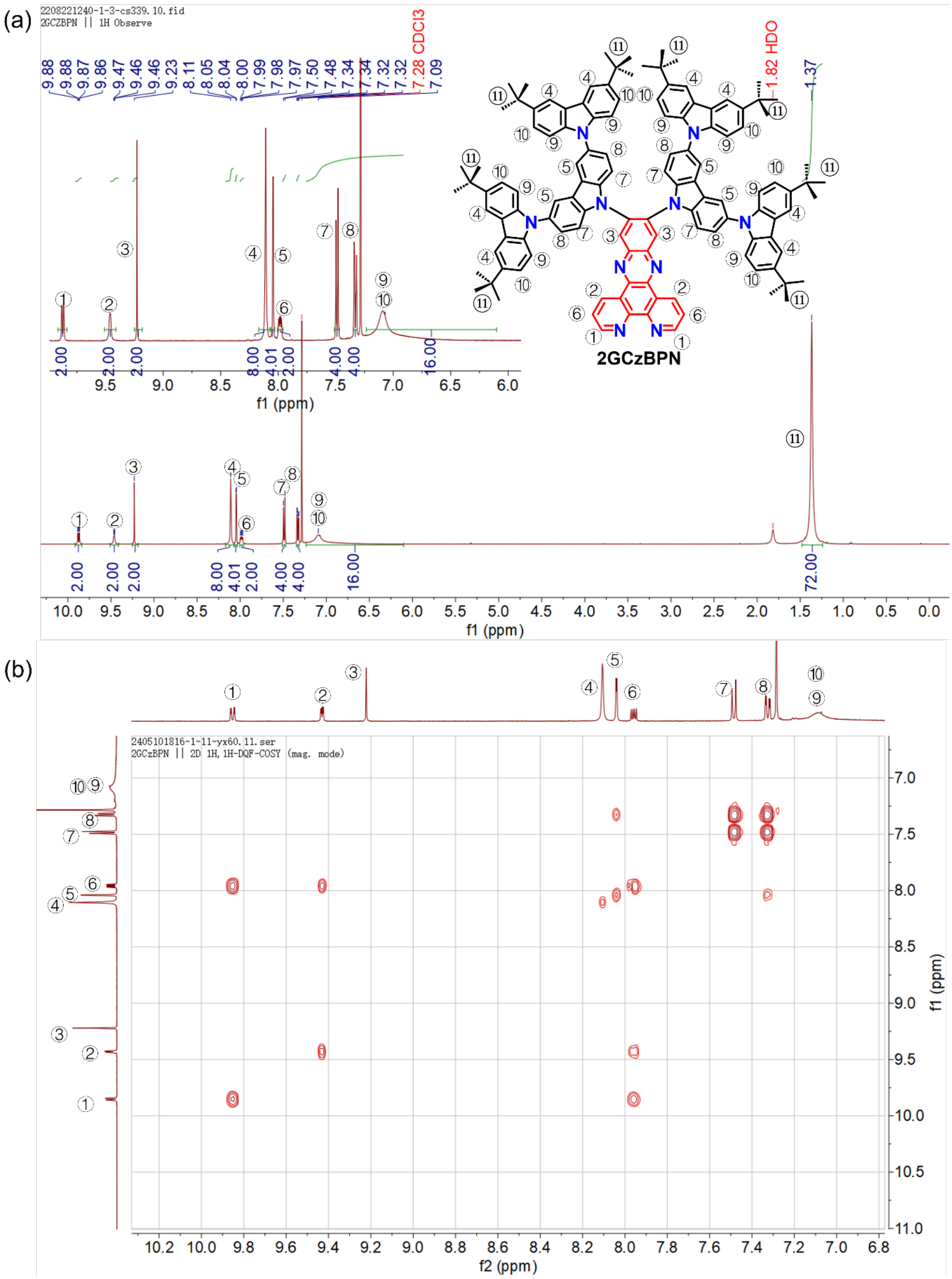

Supplementary Fig. 14. (a) 1D ^{1}H NMR and (b) ^{1}H-^{1}H COSY NMR spectra of 2GCzBPN in $CDCl_3$.

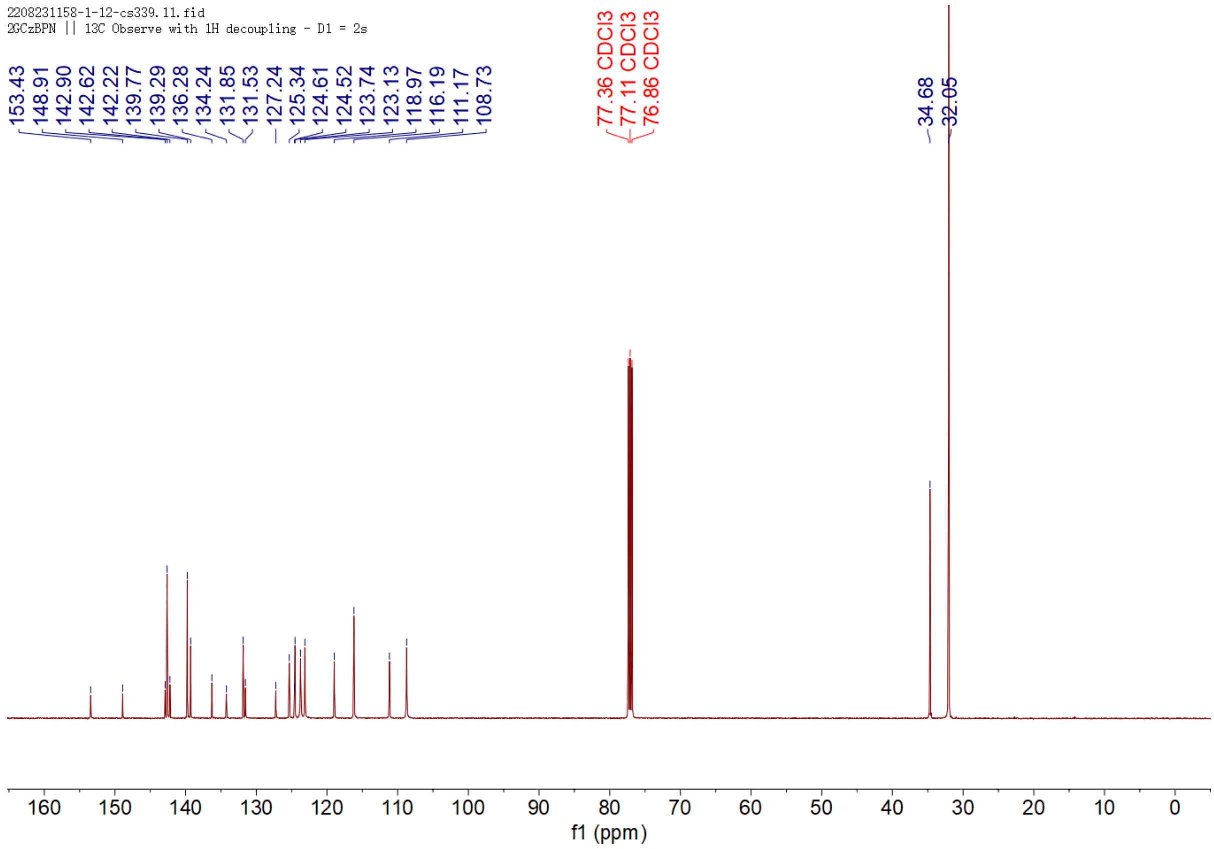

Supplementary Fig. 15. ^{13}C NMR spectra of 2GCzBPN in $CDCl_3$.

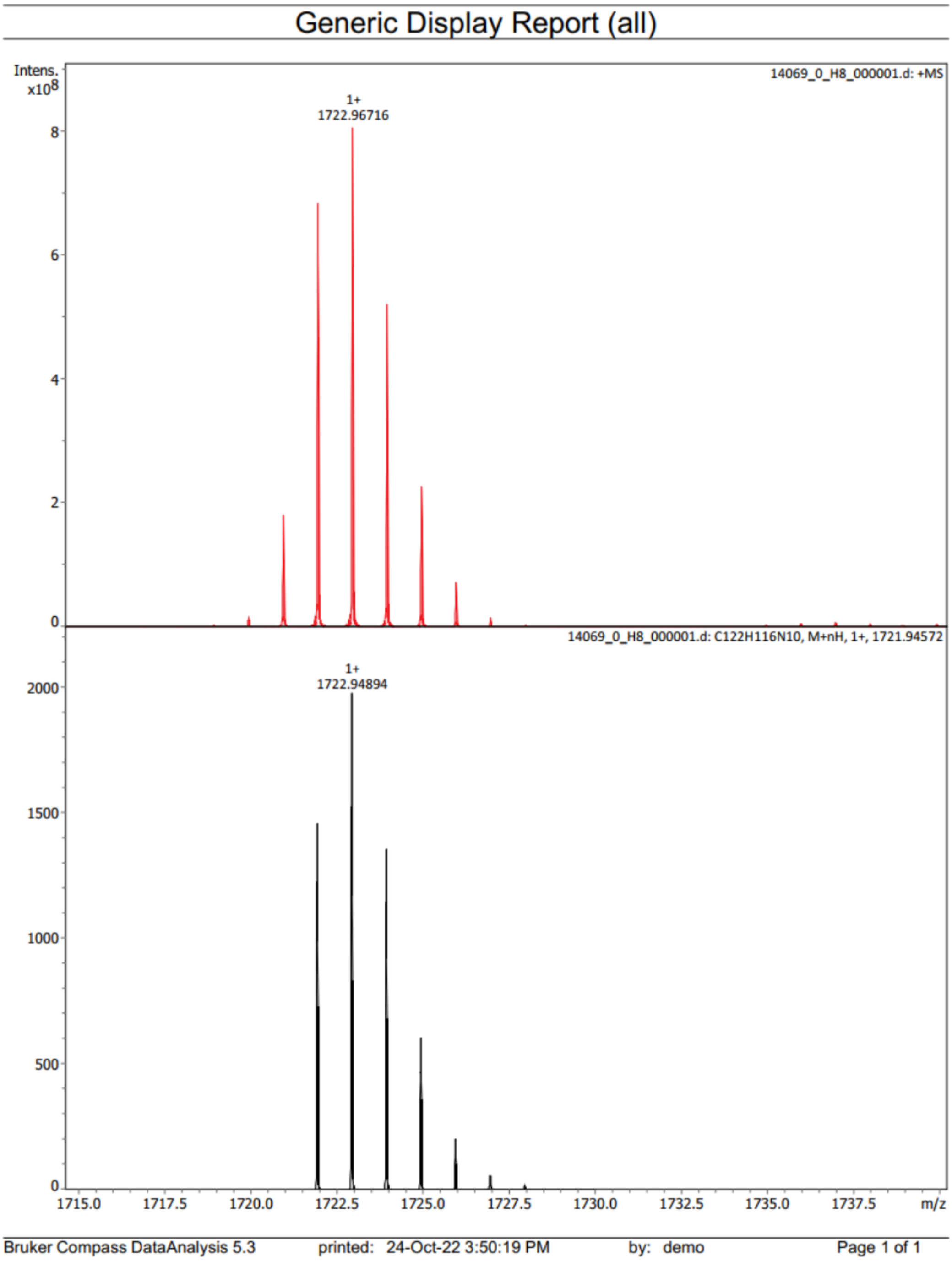

Supplementary Fig. 16. HRMS of 2GCzBPN.

Elemental Analysis Service Request Form

Researcher name Changfeng Si

Researcher email cs339@st-andrews.ac.uk

NOTE: Please submit ca. 10 mg of sample

**Sample reference number**	CS260-2GCzBPN
**Name of Compound**	2GCzBPN
**Molecular formula**	C122H116N10
**Stability**	
**Hazards**	
**Other Remarks**	

Analysis type:

Single ☐ **Duplicate** ■ **Triplicate** ☐

Analysis Result:

Element	Expected %	Found (1)	Found (2)	Found (3)
Nitrogen	8.13	8.32	8.29	
Carbon	85.08	85.76	85.23	
Hydrogen	6.79	7.04	6.96	

Authorising Signature:

**Date completed**	28.11.22
**Signature**	[illegible]
**comments**	

Supplementary Fig. 17. Elemental analysis of 2GCzBPN.

HPLC Trace Report31Mar2023

<Sample Information>

Sample Name	: 2GCZBPN		
Sample ID	:		
Method Filename	: 90% Acetonitrile 10 Water 20 mins.lcm		
Batch Filename	: BPNBP2GC.lcb		
Vial #	: 1-23	Sample Type	: Unknown
Injection Volume	: 10 uL		
Date Acquired	: 31/03/2023 21:57:50	Acquired by	: System Administrator
Date Processed	: 31/03/2023 22:17:52	Processed by	: System Administrator

<Chromatogram>

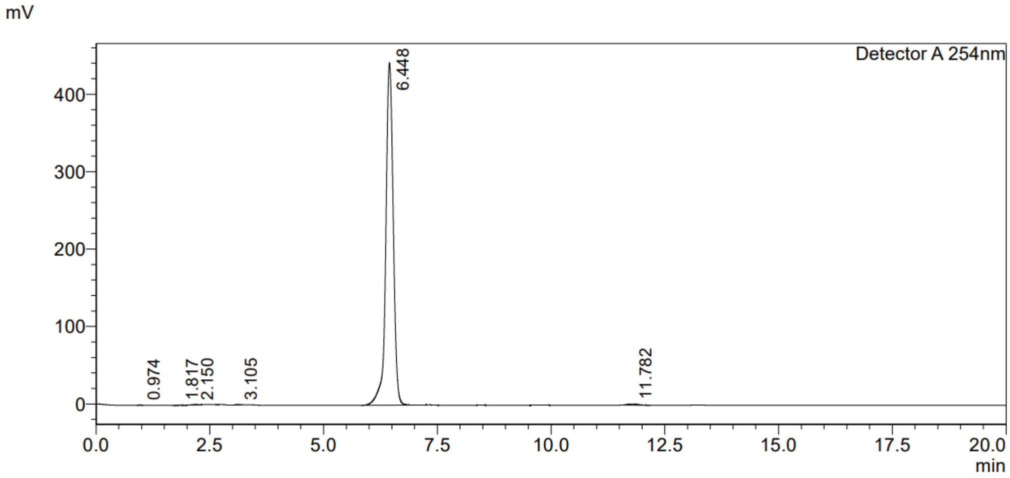

<Peak Table>

Detector A 254nm

Peak#	Ret. Time	Area	Height	Area%	Area/Height	Width at 5% Height
1	0.974	2173	637	0.043	3.412	0.099
2	1.817	2331	284	0.046	8.219	0.234
3	2.150	3258	408	0.064	7.992	0.226
4	3.105	2745	570	0.054	4.814	0.151
5	6.448	5018774	441879	99.242	11.358	0.422
6	11.782	27811	1579	0.550	17.611	0.540
Total		5057092	445357	100.000		

Supplementary Fig. 18. HPLC trace of 2GCzBPN.

Supplementary Notes

DFT Calculations

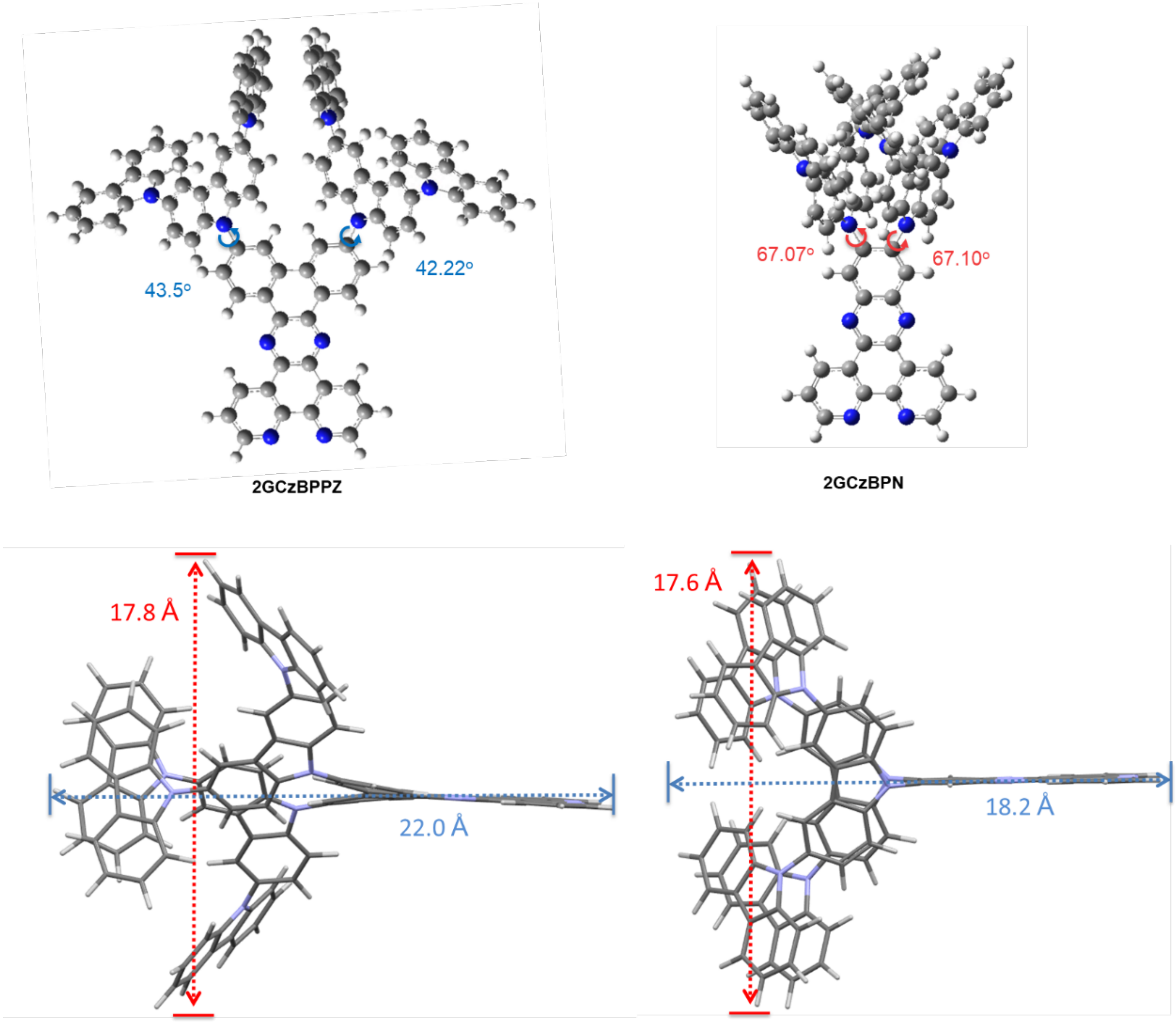

Supplementary Fig. 19. DFT-optimized molecular geometries of ground state of 2GCzBPPZ and 2GCzBPN.

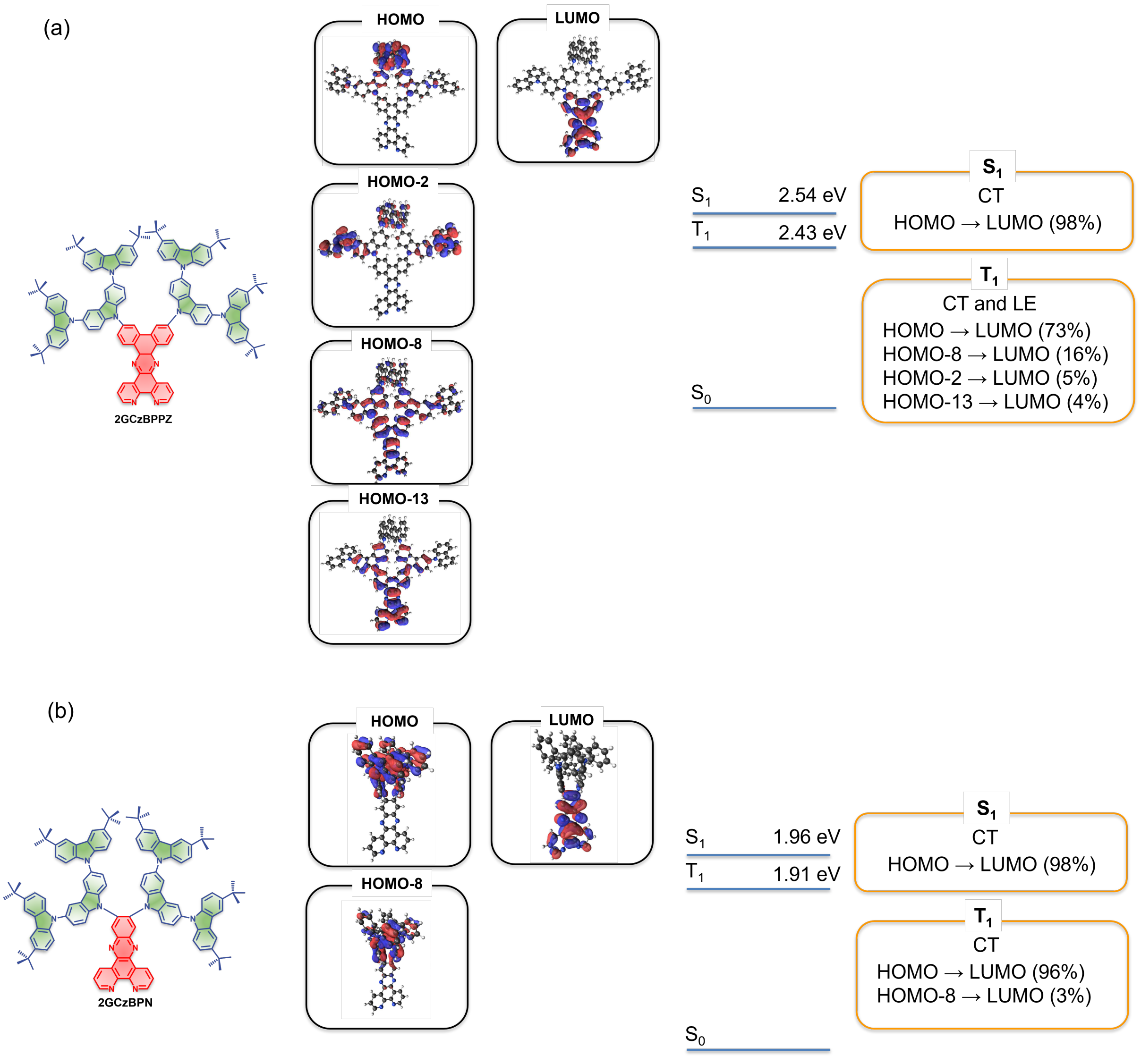

Supplementary Fig. 20. Frontier molecular orbitals, energy levels of excited states and major molecular orbital transitions of (a)2GCzBPPZ and (b) 2GCzBPN (isovalue= 0.02).

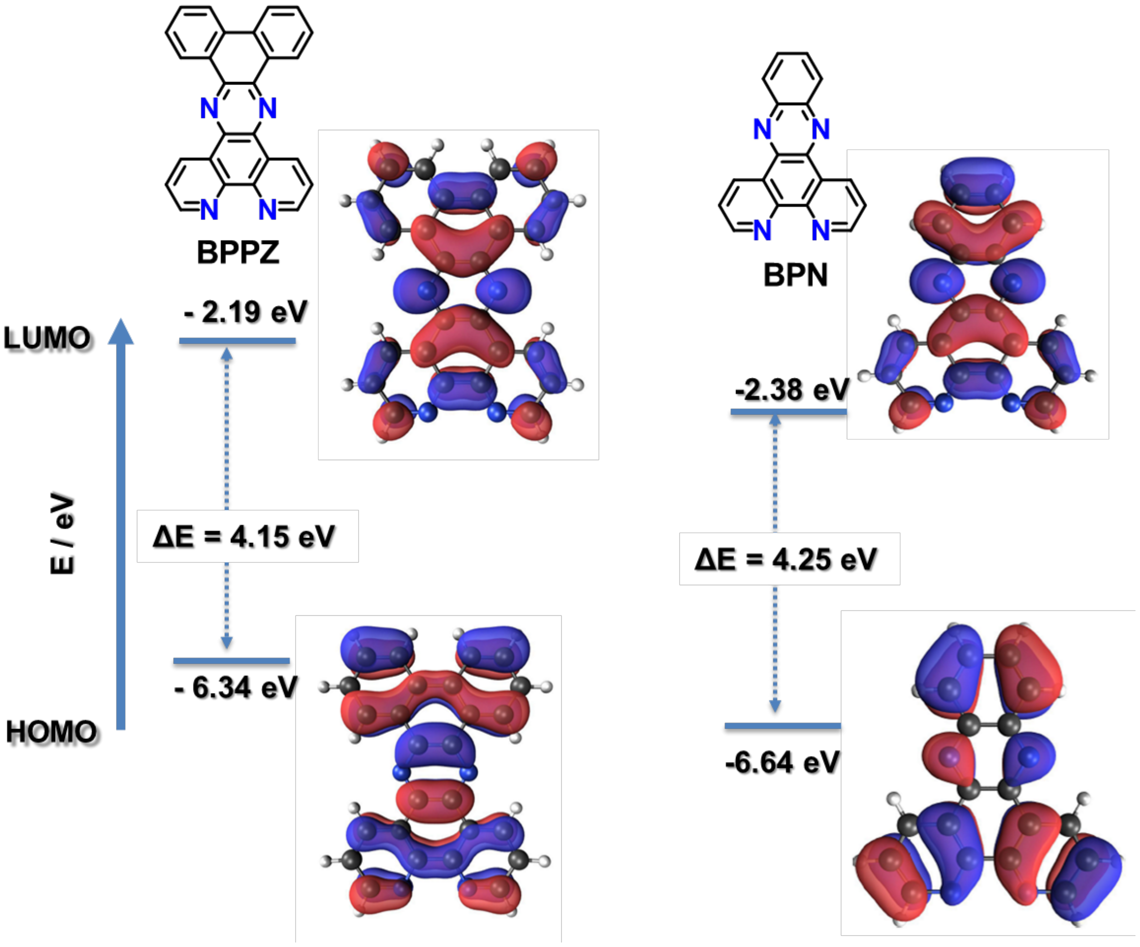

Supplementary Fig. 21. Frontier molecular orbitals (isovalue: 0.02) of the acceptors BPNBP and BPN calculated at the optimized S_0 geometry in the gas phase at the PBE0/6-31G(d,p) level.

Photophysical Properties

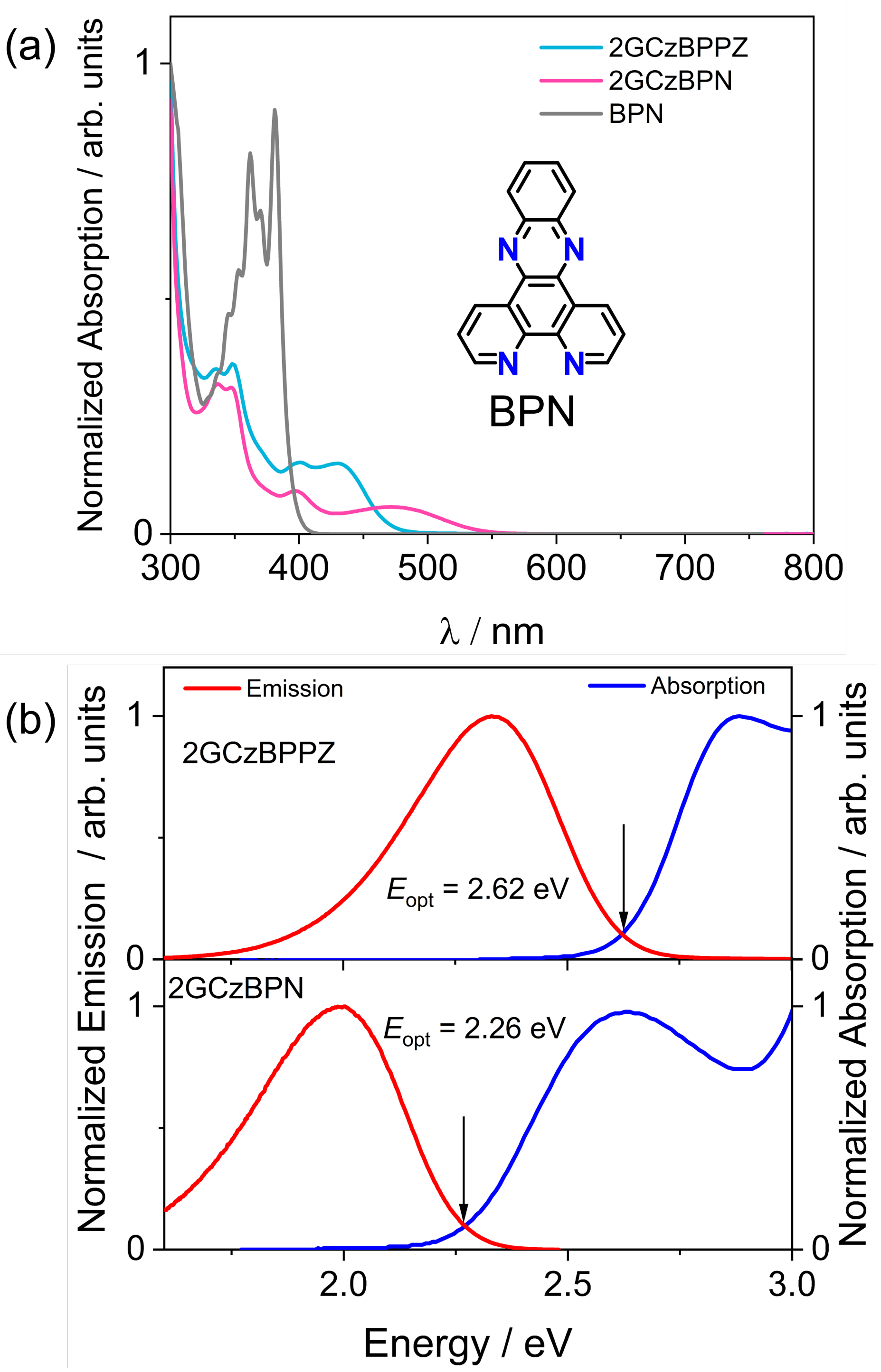

Supplementary Fig. 22. (a) Normalized UV-vis absorption for 2GCzBPPZ, 2GCzBPN and BPN in toluene; (b) The optical gaps were determined from the intersection point of the normalized absorption and emission spectra for 2GCzBPPZ and 2GCzBPN. (λ_{exc}= 343 nm).

Supplementary Table 1. Photophysical properties of 2GCzBPPZ and 2GCzBPN.

	λ_{abs}/(ε / ×10^3 M^{-1} cm^{-1})[a] / nm	λ_{PL}[a]/ nm	S_1/T_1[b]/ eV	ΔE_{ST}[b]/ eV	λ_{PL}[c]/ nm	Φ_{PL}[c] / %	τ_p[d] / ns	τ_d[d] / µs	S_1/T_1[e]/ eV	ΔE_{ST}[e] / eV	HOMO[f]/ eV	LUMO[f]/ eV	ΔE[f]/ eV
2GCzBPPZ	346 (45), 400(19), 432(19)	534	2.63/2.46	0.17	531	57 (45)	18.0	73.1	2.71/ 2.50	0.26	-5.34	-2.98	2.36
2GCzBPN	348 (51), 397(15), 475(10)	624	2.35/2.21	0.14	601	71 (60)	34.1	2.9	2.38/ 2.26	0.12	-5.26	-3.21	2.05

[a] In PhMe at 298 K (λ_{exc} = 340 nm). [b] Obtained from the onset of the prompt fluorescence (time window: 1 ns – 100 ns) and phosphorescence spectra (time window: 1 ms – 10 ms) measured in 2-MeTHF glass at 77 K, λ_{exc} = 343 nm. [c] Thin films of 10 wt% emitters doped in mCP were prepared by spin-coating, and Φ_{PL} values were determined using an integrating sphere (λ_{exc} = 345 nm). Values quoted are under N_2. Values in parentheses are in air. [d] Average lifetime ($\tau_{avg} = \Sigma A_i \tau_i^2 / \Sigma A_i \tau_i$, where A_i is the pre-exponential for lifetime τ_i). Prompt and delayed emissions were measured by TCSPC and MCS, respectively (λ_{exc} = 379 nm). [e] S_1 was obtained from the onset of the prompt emission (time window: 1–100 ns) measured in doped film at 77 K and T_1 was obtained from the onset of the phosphorescence spectrum (time window: 1–10 ms) measured in doped film at 77 K. [f] In DCM with 0.1 M [^{n}Bu$_4$N]PF$_6$ as the supporting electrolyte and Fc/Fc$^+$ as the internal reference (0.46 V vs. SCE).[3] The HOMO and LUMO energies were determined using $E_{HOMO/LUMO}$ = - (E_{ox}/ E_{red} +4.8) eV where E_{ox} and E_{red} are anodic and cathodic peak potentials versus Fc/Fc$^+$, respectively, obtained from the DPV.[21] [g] ΔE= $|E_{HOMO}$-$E_{LUMO}|$.

Regarding the calculations of transition rates, the related equations were used as follows:[4,5]

$$k_{\mathrm{p}}=\frac{1}{\tau_{\mathrm{p}}} \tag{1}$$

$$k_{\mathrm{d}}=\frac{1}{\tau_{\mathrm{d}}} \tag{2}$$

$$k_{\mathrm{r}}=k_{\mathrm{p}}\Phi_{\mathrm{p}} \tag{3}$$

$$k_{\mathrm{ISC}}=k_{\mathrm{r}}\times(1-\Phi_{\mathrm{p}}) \tag{4}$$

τ_{p}: prompt lifetime;

τ_{d}: delayed lifetime;

k_{p}: prompt fluorescence component;

k_{d}: delayed fluorescence component;

k_{r}: radiative decay rate constant;

k_{ISC}: intersystem crossing rate constant;

Φ_{p}: prompt emission efficiency;

Φ_{d}: delayed emission efficiency;

k_{RISC} (reverse intersystem crossing rate constant) was calculated according to the equation:

$$k_{\mathrm{RISC}}=\frac{k_{\mathrm{p}}k_{\mathrm{d}}}{k_{\mathrm{ISC}}}\frac{\Phi_{\mathrm{d}}}{\Phi_{\mathrm{p}}} \tag{5}$$

Where $\boldsymbol{\Phi_d}$ and $\boldsymbol{\Phi_p}$are the PLQY of delayed and prompt components, respectively.

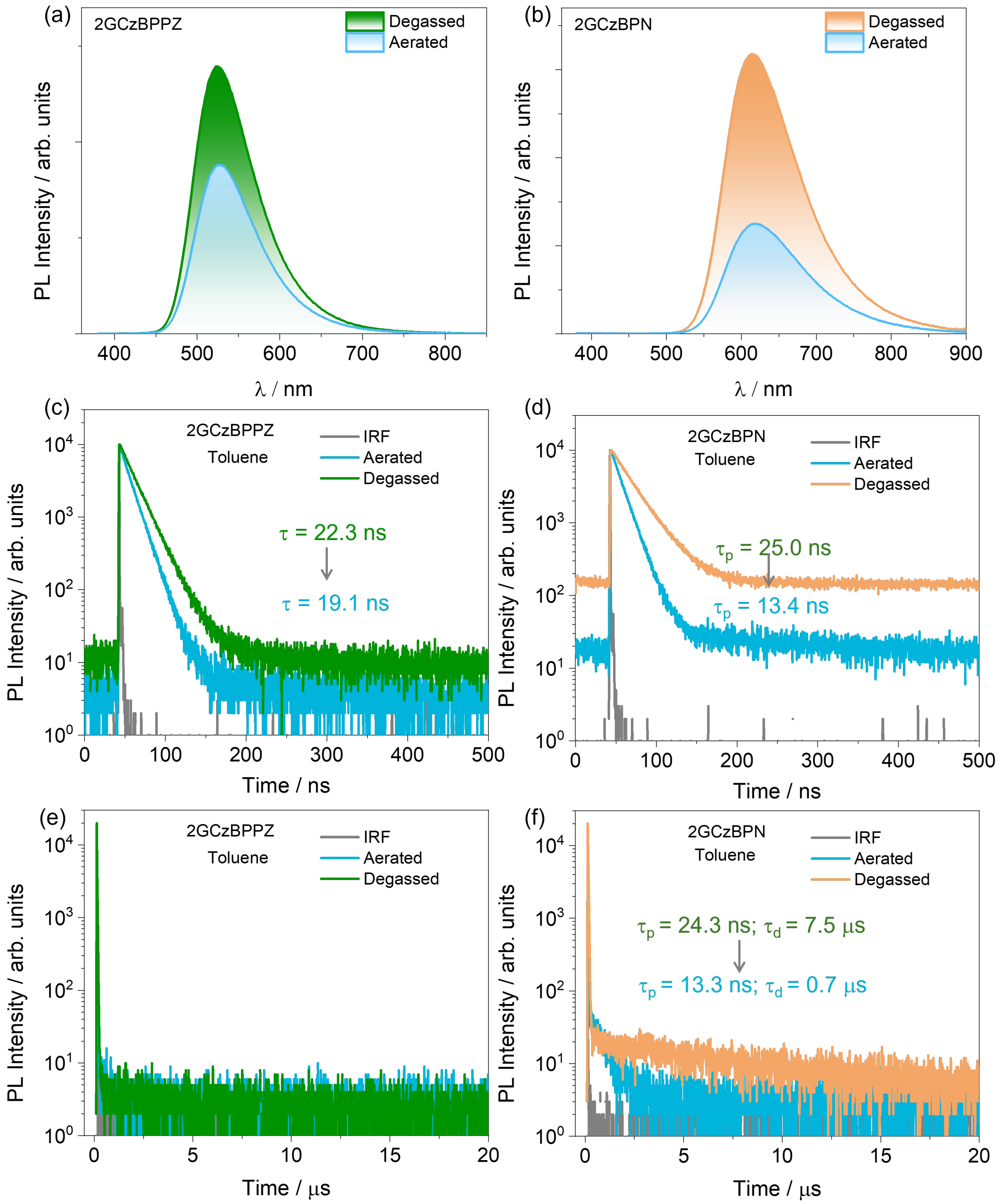

Supplementary Fig. 23. Steady-state PL spectra of (a) 2GCzBPPZ, (b) 2GCzBPN, in degassed and aerated toluene (λ_{exc}= 343 nm, at a concentration of 1.6×10^{-5} M). Time-resolved PL decay profiles of (c) 2GCzBPPZ, (d) 2GCzBPN with time window 0-500 ns and (e) 2GCzBPPZ, (f) 2GCzBPN with time window 0-20 μs using TCSPC in aerated and degassed toluene (λ_{exc}= 379 nm).

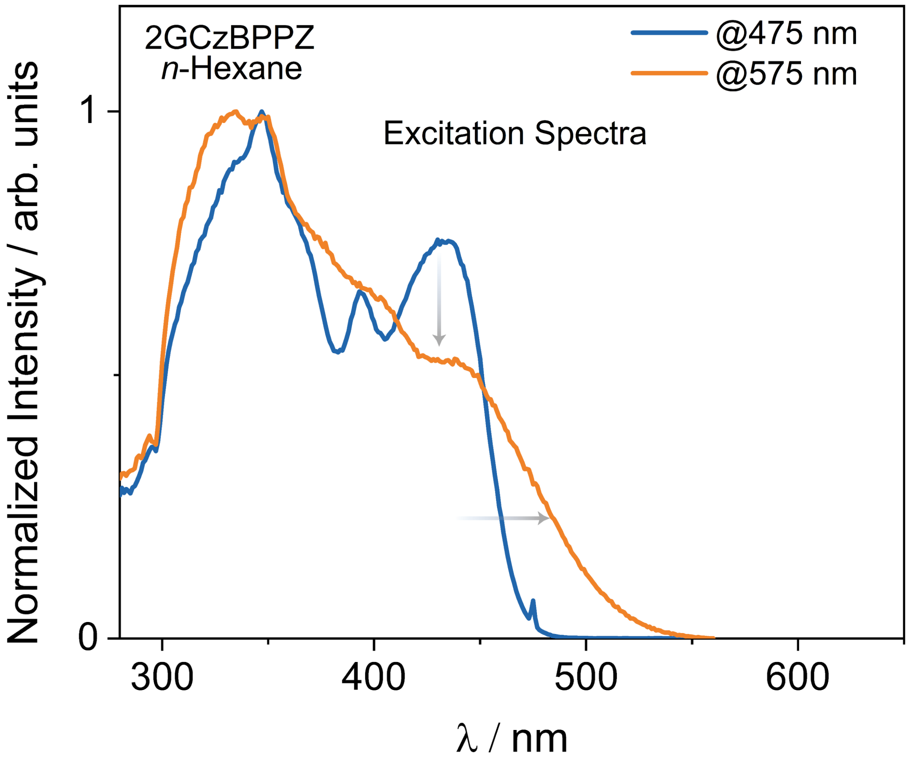

Supplementary Fig. 24. Excitation spectra of 2GCzBPPZ recorded at λ_{PL} 475 and 575 nm in n-hexane at 298 K; concentration: 2×10^{-5} M.

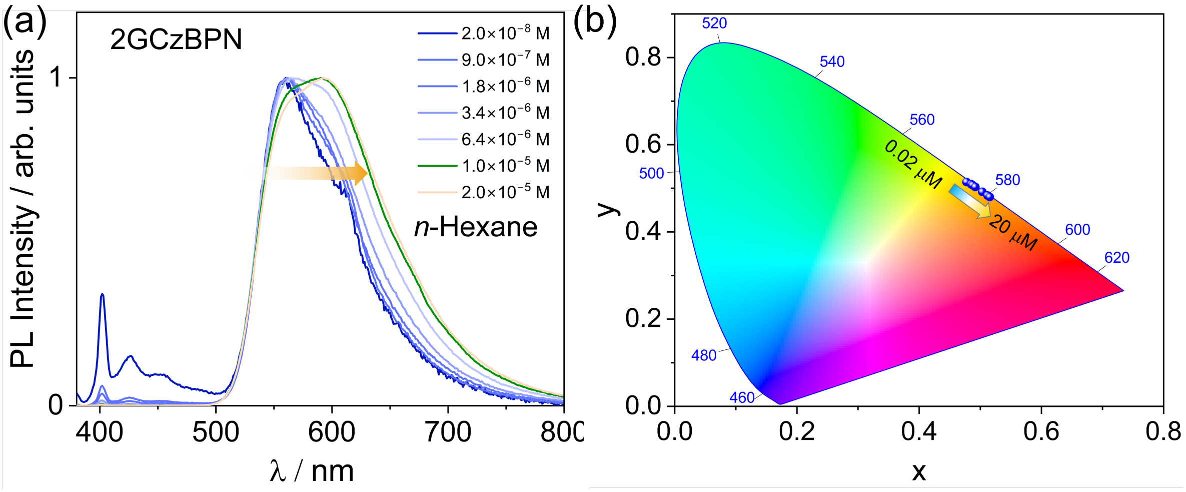

Supplementary Fig. 25. (a) Concentration-dependent emission spectra for 2GCzBPN in n-hexane solution (λ_{exc} = 340 nm); (b) CIE plot of the color evolution of 2GCzBPN as a function of concentration.

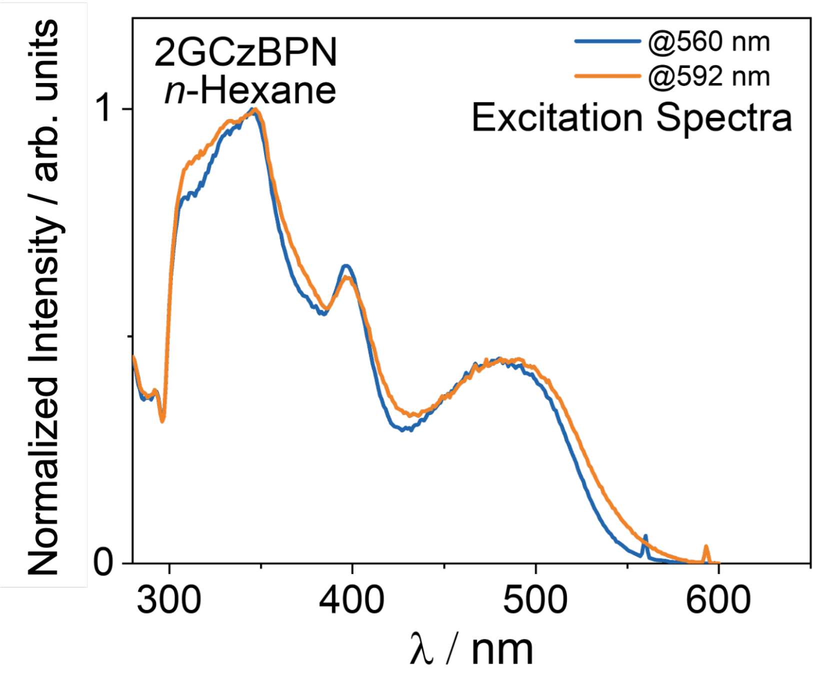

Supplementary Fig. 26. Excitation spectra of 2GCzBPN recorded at λ_{PL} 560 and 592 nm in *n*-hexane at 298 K.

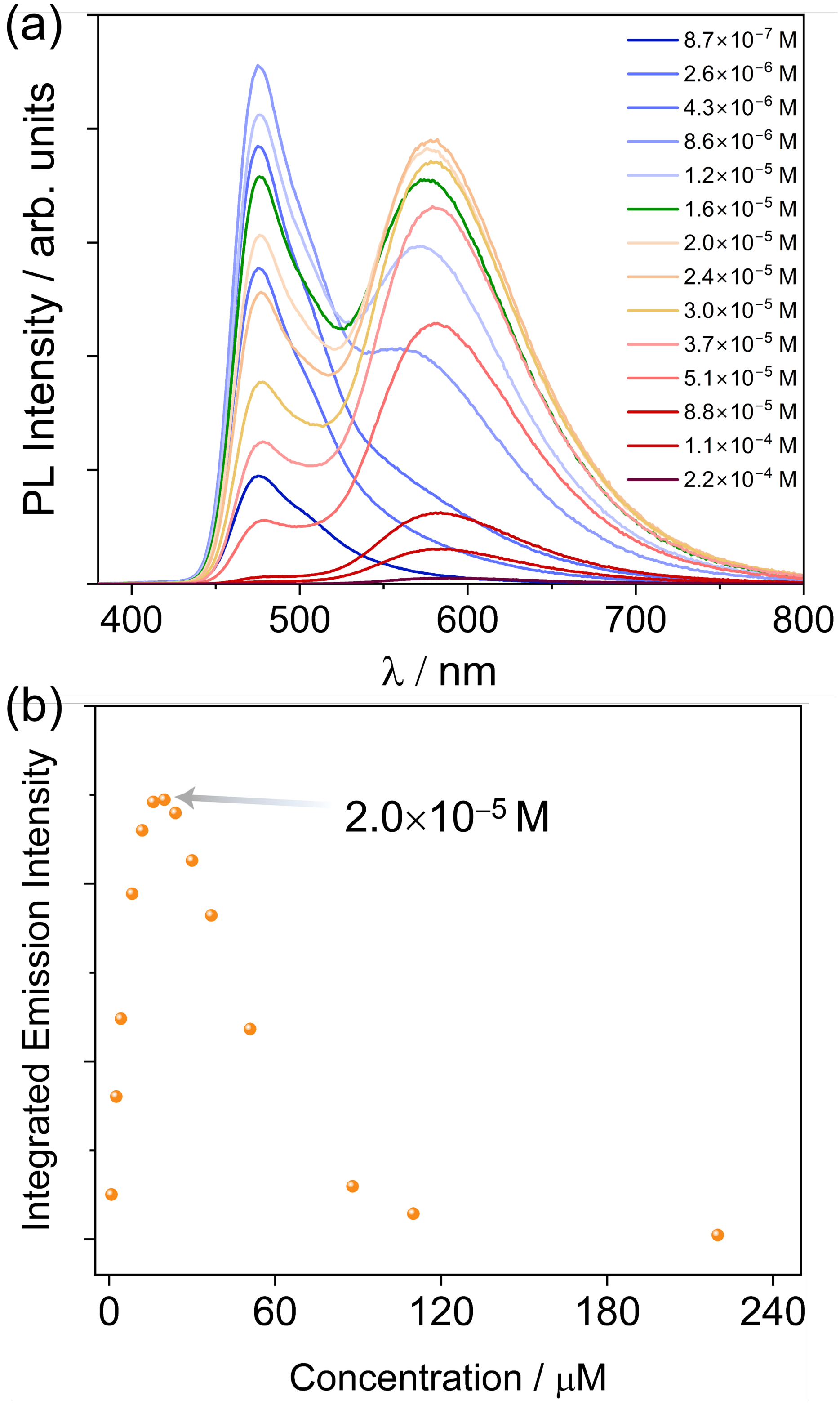

Supplementary Fig. 27. (a) Concentration-dependent PL spectra for 2GCzBPPZ in *n*-hexane and (b) the corresponding integrated emission intensity at different concentrations of 2GCzBPPZ in *n*-hexane.

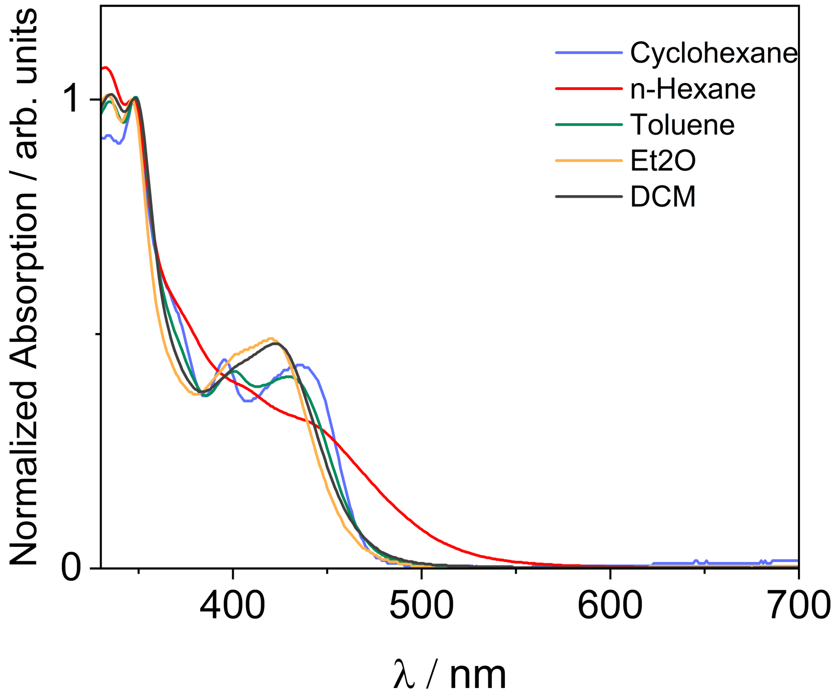

Supplementary Fig. 28. Absorption spectra for 2GCzBPPZ in cyclohexane, *n*-hexane, toluene, Et_2O, and DCM at the concentration of 2×10^{-5} M. All absorption spectra are normalized at the 348 nm peak, which is associated with the absorption of the donor GCz).

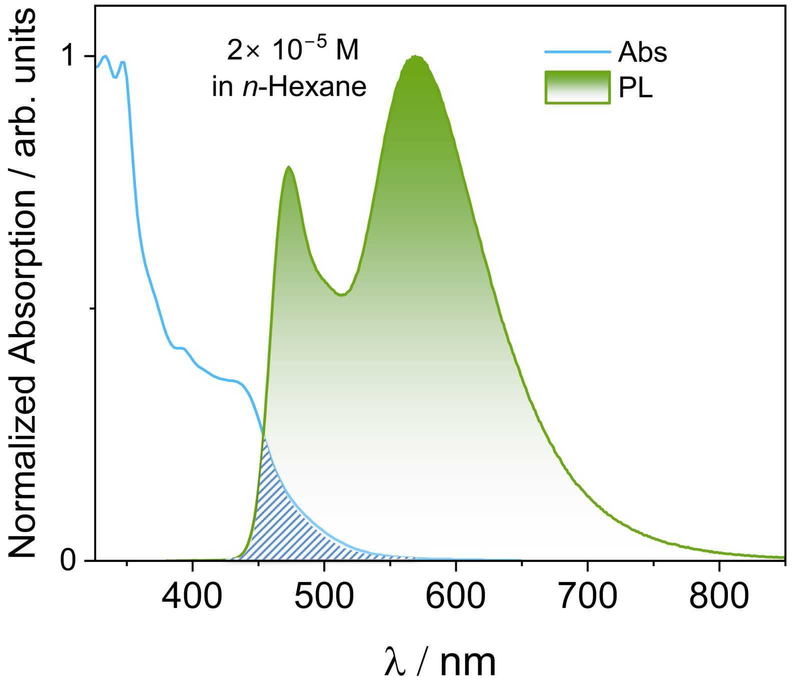

Supplementary Fig. 29. Absorption and PL spectra for 2GCzBPPZ in *n*-hexane at the concentration of 2 × 10^{-5} M. The spectral overlap favors the FRET between the monomer emission and aggregate absorption.

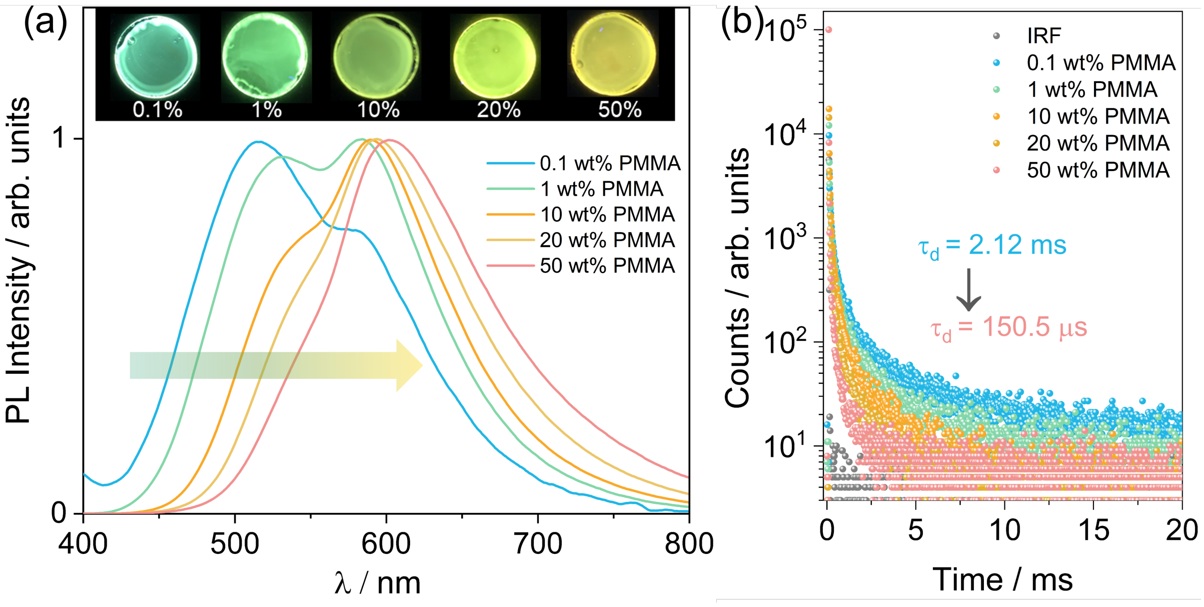

Supplementary Fig. 30. (a) Normalized steady-state PL spectra of increasing doping concentrations (from 0.1 wt% to 50 wt%) for 2GCzBPPZ in PMMA in vacuum (λ_{exc} = 340 nm); (b) PL decays of the doped films in PMMA (λ_{exc} = 340 nm).

Supplementary Table 2. Φ_{PL} values in the film state and toluene.

Emitter	Host	Doping ratio	Φ_{PL} / % (in N_2)	Φ_{PL} / % (in air)
2GCzBPPZ	PMMA[a]	0.1 wt%	32	26
		1 wt%	72	55
		10 wt%	37	42
		20 wt%	31	27
		50 wt%	29	27
		neat	11	10
	Toluene[b]	-	45 (degassed)	31 (aerated)

[a]Thin films of the 2GCzBPPZ in PMMA at different doping concentrations were prepared by spin-coating. The Φ_{PL} values of the thin films were measured using an integrating sphere (λ_{exc} = 350 nm) in air or a N_2 at 298 K. [b] Quinine sulfate in H_2SO_4 (aq) was used as the reference (Φ_{PL} = 54.6%, λ_{exc}=360 nm) for the solution-state measurements.[6] Φ_{PL} for degassed solutions, which were prepared by three freeze-pump-thaw cycles. Φ_{PL} for aerated solutions were prepared by bubbling air for 10 min.

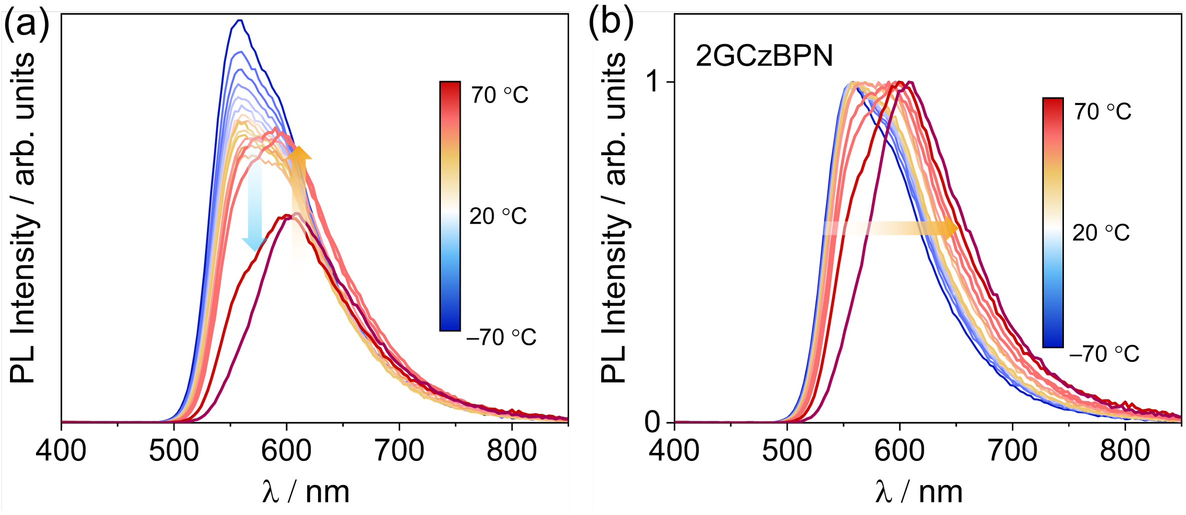

Supplementary Fig. 31. (a) Temperature-dependent PL spectra and (b) normalized PL of 2GCzBPN in *n*-hexane at a concentration of 1 × 10^{-5} M (λ_{exc} = 340 nm).

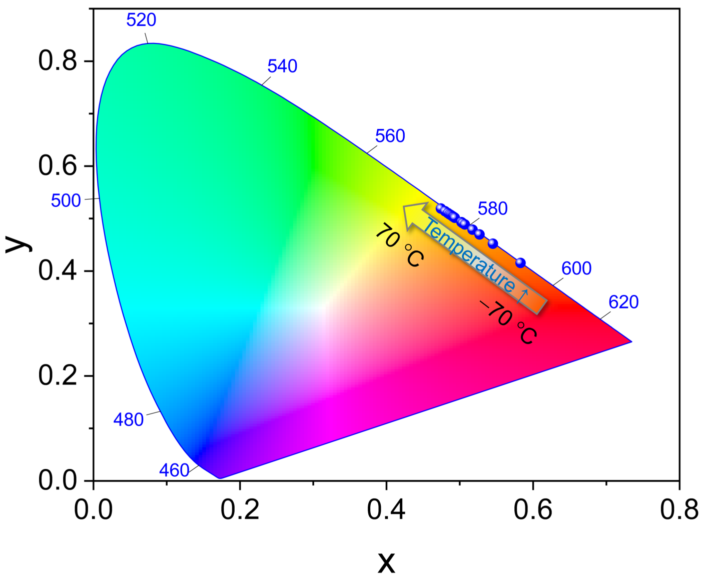

Supplementary Fig. 32. CIE plot of the temperature-dependent emission spectra of 2GCzBPN in *n*-hexane at a concentration of 1 × 10^{-5} M (λ_{exc} = 340 nm).

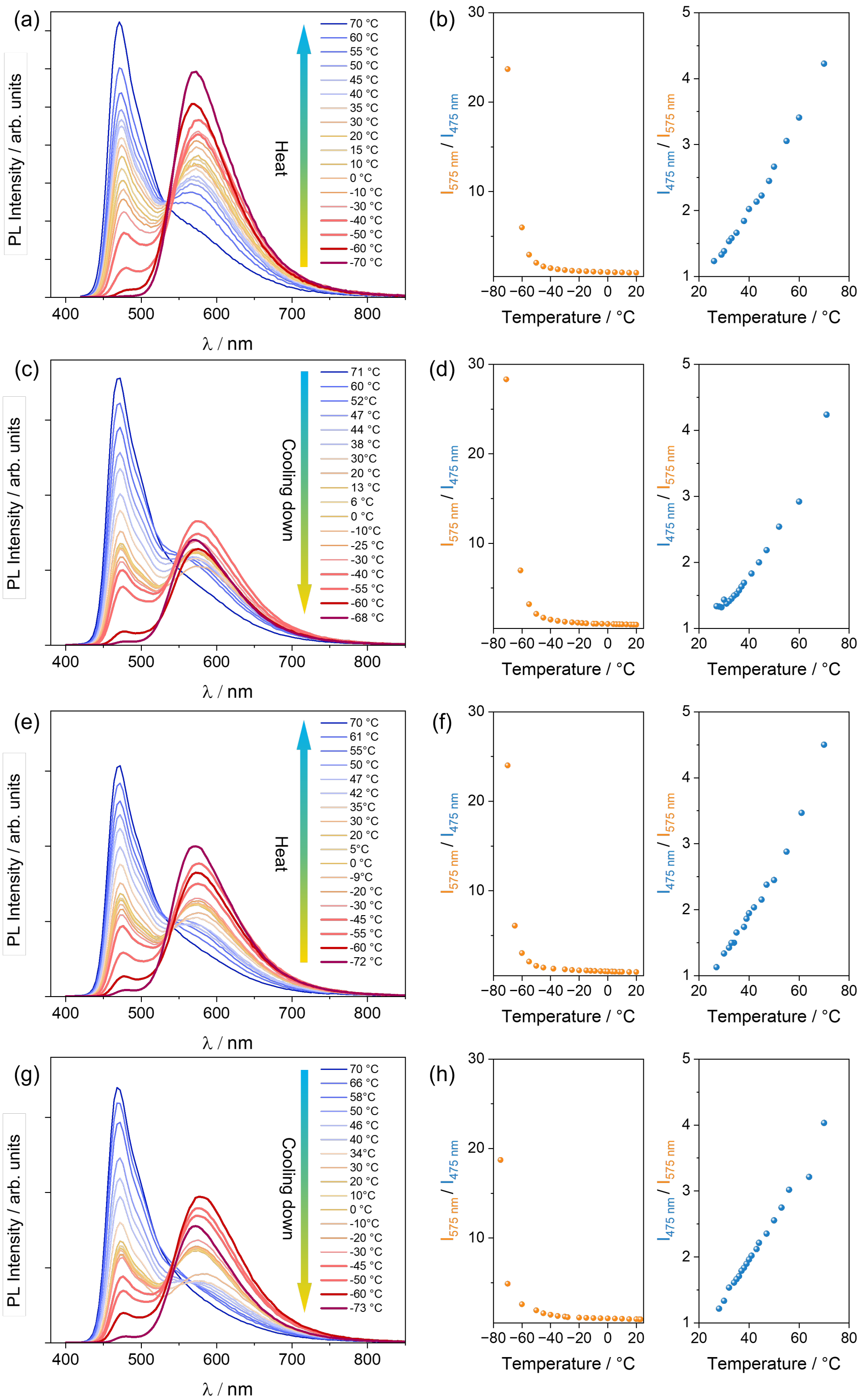

Supplementary Fig. 33. Temperature dependent PL spectra (a, c, e, and g) and the ratiometric plot of I_{475}/I_{575} and I_{475}/I_{575} (b, d, f, and h) versus temperature in four cycles based on 2GCzBPPZ. (a) First cycle. (c) Second cycle. (e) Third cycle. (g) Fourth cycle (λ_{exc} = 340 nm).

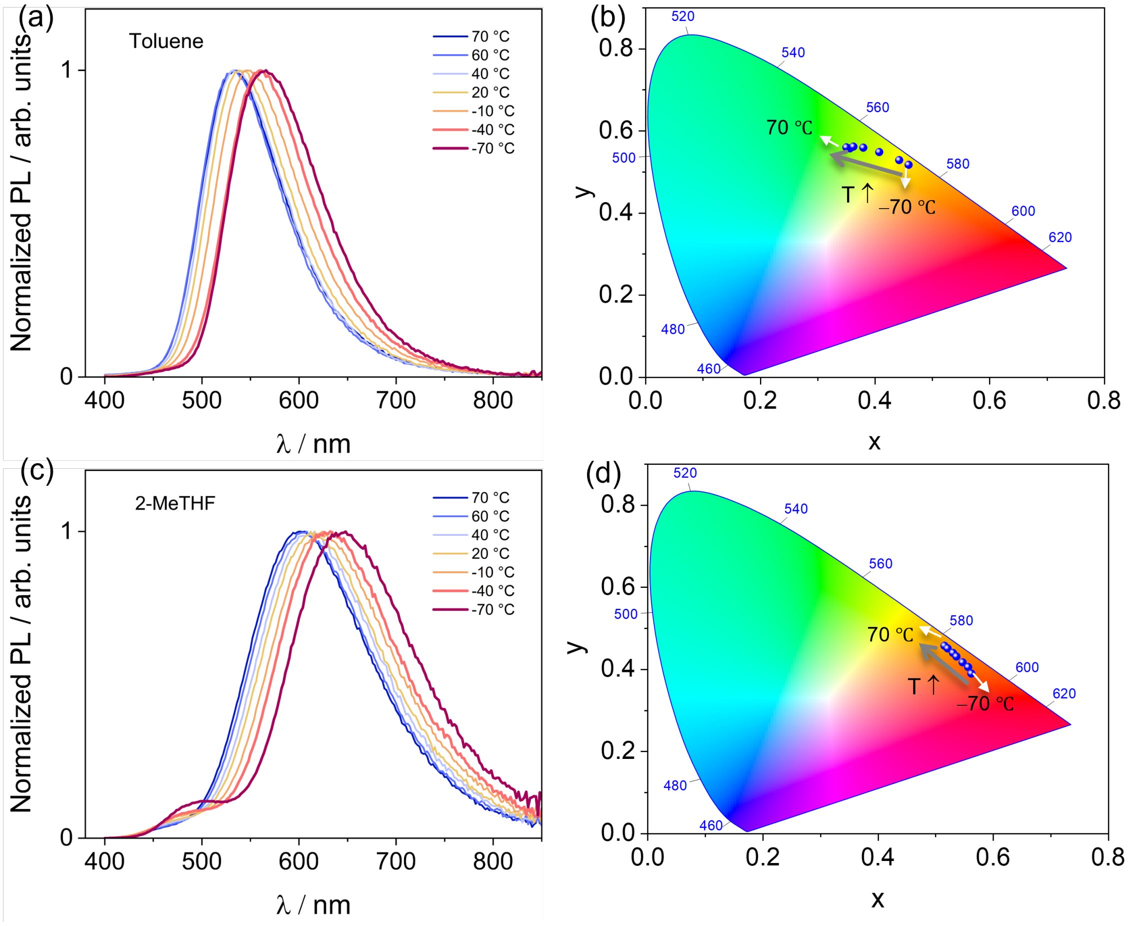

Supplementary Fig. 34. (a, c) Temperature-dependent steady-state PL spectra from -70 to 70 °C and (b, d) CIE diagrams of 2GCzBPPZ in (a, b) toluene and (c, d) 2-MeTHF, respectively, at a concentration of 1.6×10^{-5} M ($\lambda_{exc} = 340$ nm).

It seems unreliable to deduce an activation energy barrier in our system considering that the mechanism involves temperature-regulated distance changes between two monomers in the aggregate, which thus also influences the energy transfer efficiency between monomer and aggregate, thereby affecting the relative intensity of the PL from each species. Here, we estimated the temperature-dependent FRET efficiency based on Figure 5a and Supplementary Fig. 35 by deconvoluting the emission from the two components using the equation:

$$E = 1 - F_m/F_a$$

where E, F_m, and F_a represent the FRET efficiency, emission integral intensities of monomer and aggregates, respectively. These data are presented in Supplementary Table 3 and Supplementary Fig. 31, clearly demonstrating a weakened FRET efficiency attributed to an increased acceptor distance in the aggregate and thus fewer aggregates at elevated temperatures.

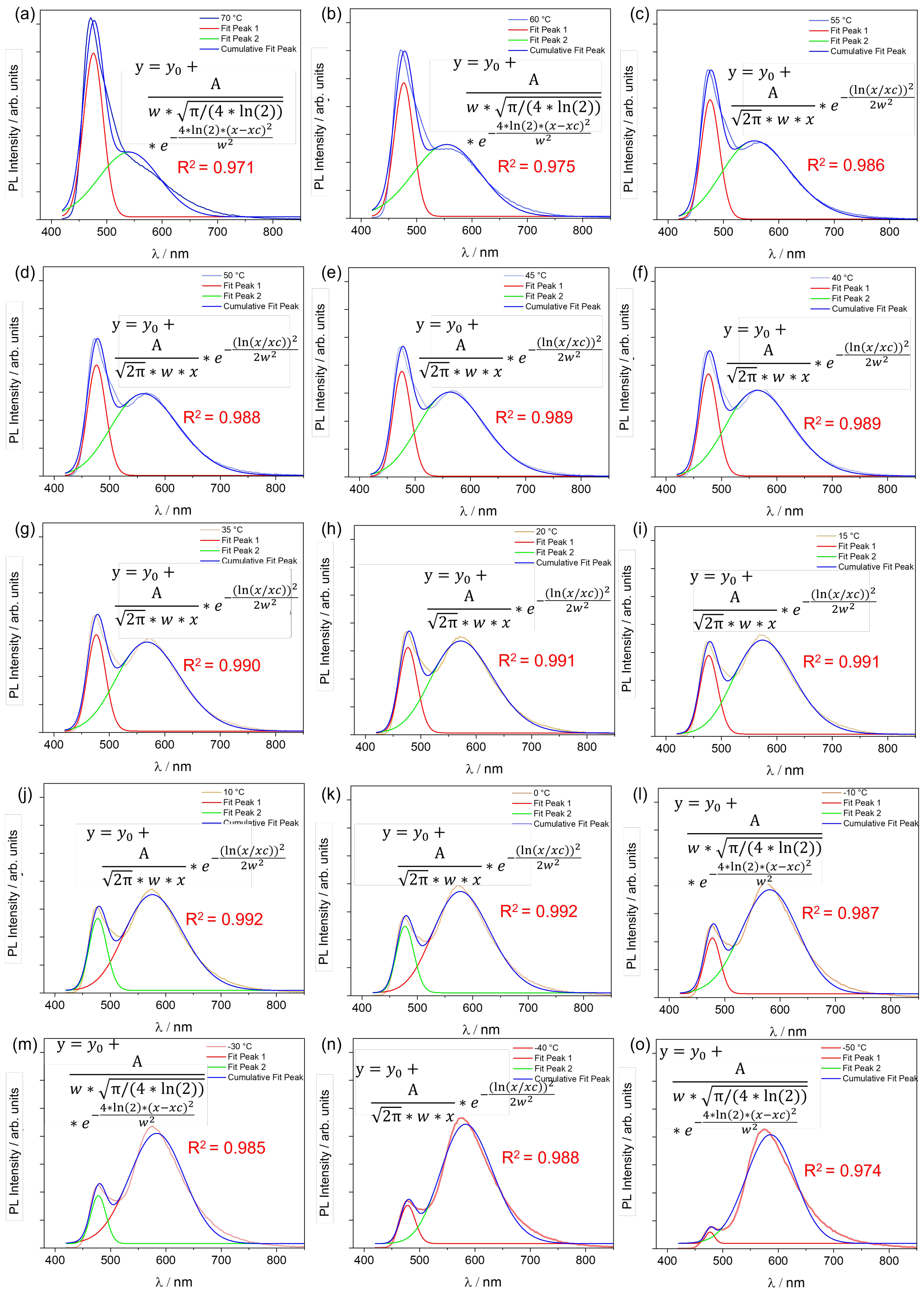

Supplementary Fig. 35. Deconvoluted emission spectra at (a) 70 °C, (b) 60 °C, (c) 55 °C, (d) 50 °C, (e) 45 °C, (f) 40 °C, (g) 35 °C, (h) 20 °C, (i) 15 °C, (j) 10 °C, (k) 0 °C, (l) –10 °C, (m) –30 °C, (n) –40 °C and (o) –50 °C (λ_{exc} = 340 nm).

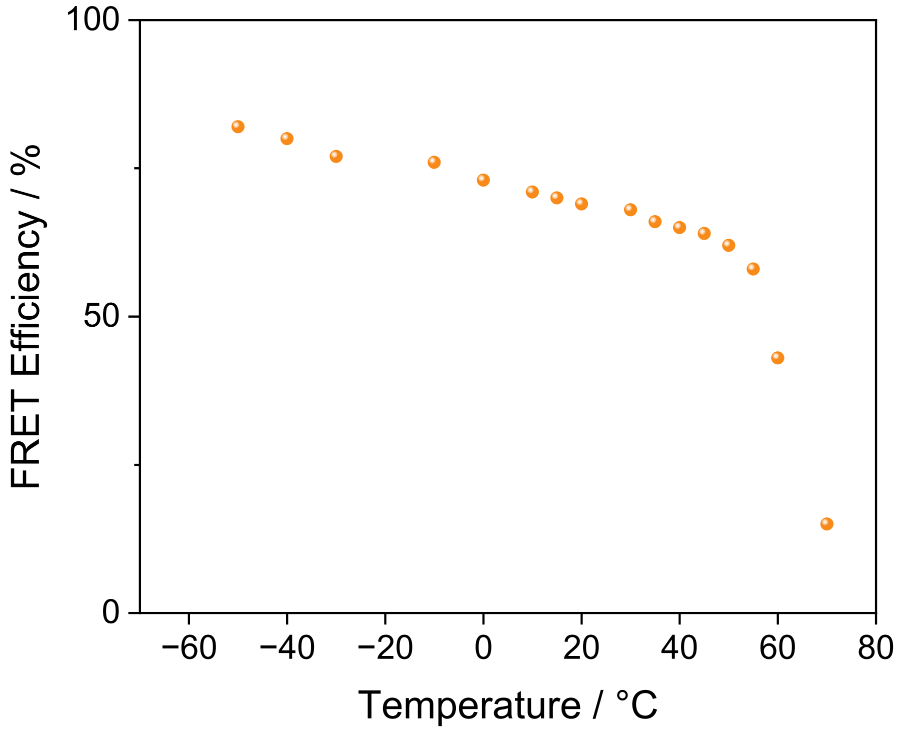

Supplementary Fig. 36. FRET efficiency as a function of temperature.

Supplementary Table 3. Summary of the FRET efficiency as a function of temperature.

Temperature / ℃	F_m / a.u.	F_a/a.u.	FRET efficiency (E) / %
70	6035198	7088949	15%
60	4682818	8244010	43%
55	3719588	8791500	58%
50	3473600	9094263	62%
45	3317975	9303509	64%
40	3303105	9380640	65%
35	3237075	9597493	66%
30	3154777	9789308	68%
20	3036027	9719204	69%
15	2924883	9762602	70%
10	2819091	9820884	71%
0	2753592	10198878	73%
-10	2622594	10771734	76%
-30	2544623	11145801	77%
-40	2277048	11403586	80%
-50	1804586	10279165	82%
-60	-	-	-
-70	-	-	-

Supplementary Table 4. Summary of temperature sensing performance of reported and our work.

Compound Name	Emission type	Single Compound	States	Response type	λ_{PL} / nm	Color Change	Working range / °C	Relative Sensitivity / °C^{-1}	Ref.
TADF emitter									
2GCzBPPZ	monomer +aggregates	Yes	Solution	Ratio	475/575	yellow, white, blue	–70-70 °C	6.6%	This Work
	monomer +aggregates	Yes	Solid + Solution (Paraffin)	Ratio	500/570	yellow, green, blue	20-200 °C	~1.5%	
Poly(NAI-DMAC): tBuODA	Host + dopant	No	Solution	Ratio	390, 660	red to blue	35-70 °C	32%	7
TADF emitter doped in P(VDC-co- AN)	Host + dopant	No	Solid	Lifetimes	-	no	5–50 °C	4.2 %	8
C_{70} in polymer NPs	Host + dopant	No	Solid	Intensity	~670	no	–75-105 °C	-	9
Phosphorescent emitters									
PXZ-Nap	Host + dopant	no	Solid	Ratio	475-530	blue to green	–196-22 °C	-	10
TPXZPhCor	Host + dopant	no	Solid	Ratio	530-582	Green to Orange	–196-22 °C	-	11
Br6A	Host + dopant	no	Solid	Intensity	520 nm	no	30−60 °C		12
Ph-C8Br	Fl+ Ph	yes	crystal	Ratio	430/506	off-white, yellow to green	–193-0 °C	-	13
p-DFFM	Ph	yes	crystal	Lifetimes	550	no	–10 - 50 °C	-	14

Fluorescent emitters									
BAI	ICT	yes	Solution	Intensity	559	green	10 -50 ℃	2.4%	[15]
Triarylphosphine oxide	LE+CT	yes	Solution	Ratio	380/480	Blue to green	−50-100 ℃	2.4%	[16]
FIPAC	configuration transformations	yes	Solution	Ratio	442/595	orange, magenta, blue	−135-70℃	-	[17]
Tetrahydropyrimidine	Packing	yes	Solid	Ratio	434/510	Blue -green	−196-50 ℃	19.4%	[18]
6-FAM, TAMRA labeled ssDNAs	Host + dopant	no	Solid	Ratio	515/575	Green to yellow	0-100 ℃	7%	[19]
MPB-PEG4000	ICT+TICT	yes	Solid	Ratio	430,530	Blue -green	−70-100 ℃	1.4%	[20]
DB-TPE nanoparticles	Host + dopant	no	NPs	On-off	479	white	−5-65 ℃	1.1%	[21]
1,4-bis-p-cyanostyrylbenzene	Packing	no	nano-cocrystal	Ratio	465, 494,532	Blue to green	30-240 ℃	-	[22]

NPs: nanoparticles, Fl: Fluorescence, Ph: Phosphorescence, CT: Charge Transfer, ICT: Intramolecular Charge Transfer, TICT: Twisted Intramolecular Charge Transfer, LE: Locally-Excited state

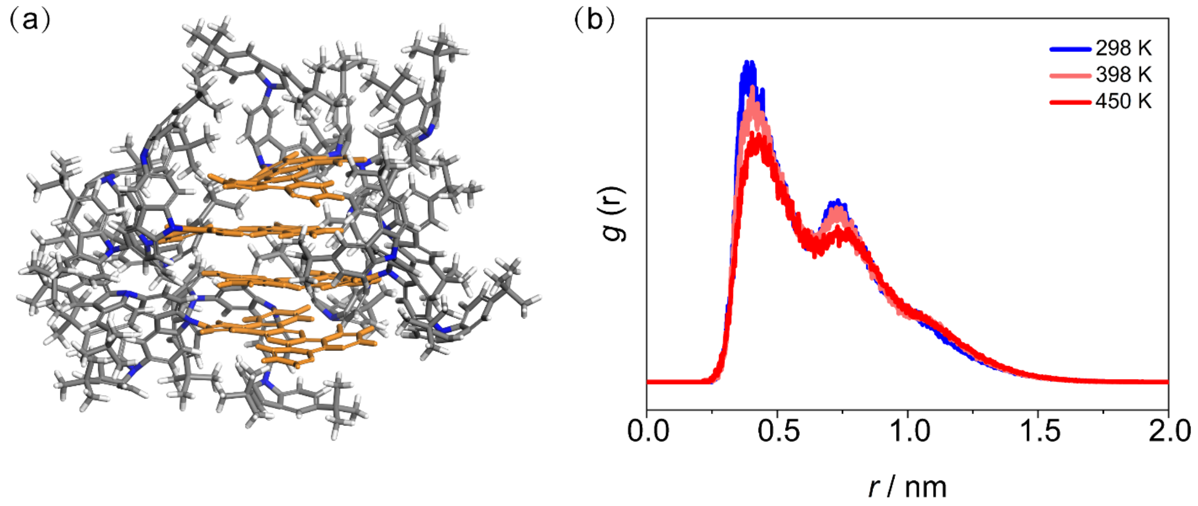

Supplementary Fig. 37. (a) Optimized geometry of 2GCzBPPZ employed for the MD simulation. (b) Radial distribution function calculated as a function of the distance between acceptors (highlighted in orange) in the aggregate. The increase in g (r) at the distance of 0.38 nm as temperature decreases indicates the distance increase between adjacent acceptors in the aggregate with increasing temperature.

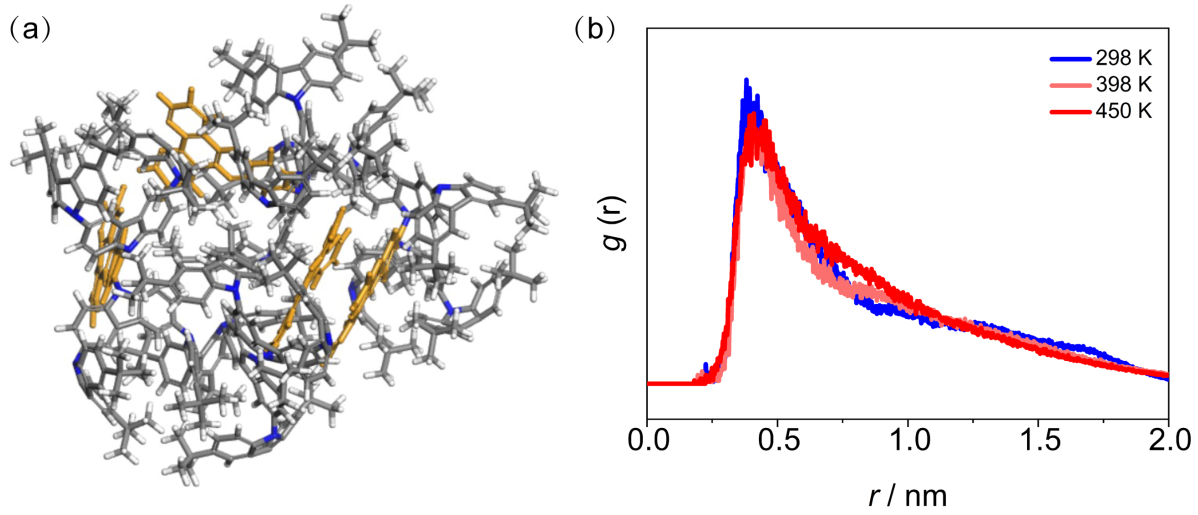

Supplementary Fig. 38. (a) Optimized geometry of 2GCzBPN employed for the MD simulation. (b) Radial distribution function calculated as a function of the distance between acceptors (highlighted in orange in a) in the aggregate. The simulation results demonstrate a negligible temperature-responsive change in the packing as the radial distribution function g(r) does not change significantly with temperature.

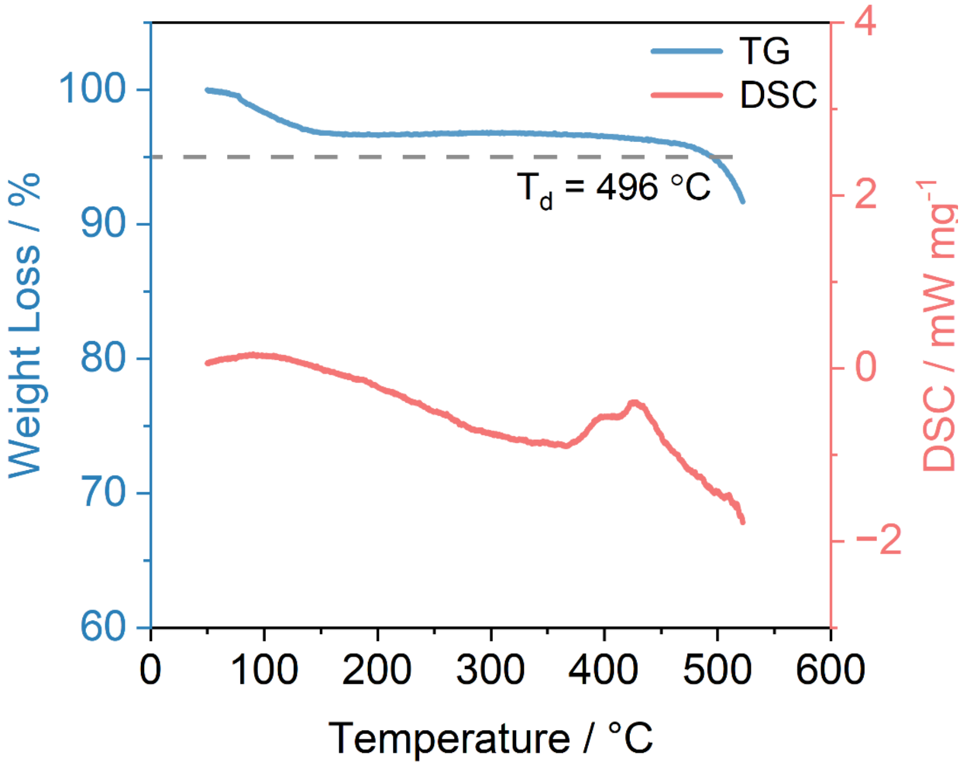

Supplementary Fig. 39. TGA/DSC results of 2GCzBPPZ. The measurement was performed with continuous N_2 and at a temperature rising rate of 10 C/min from room temperature to 550 °C.

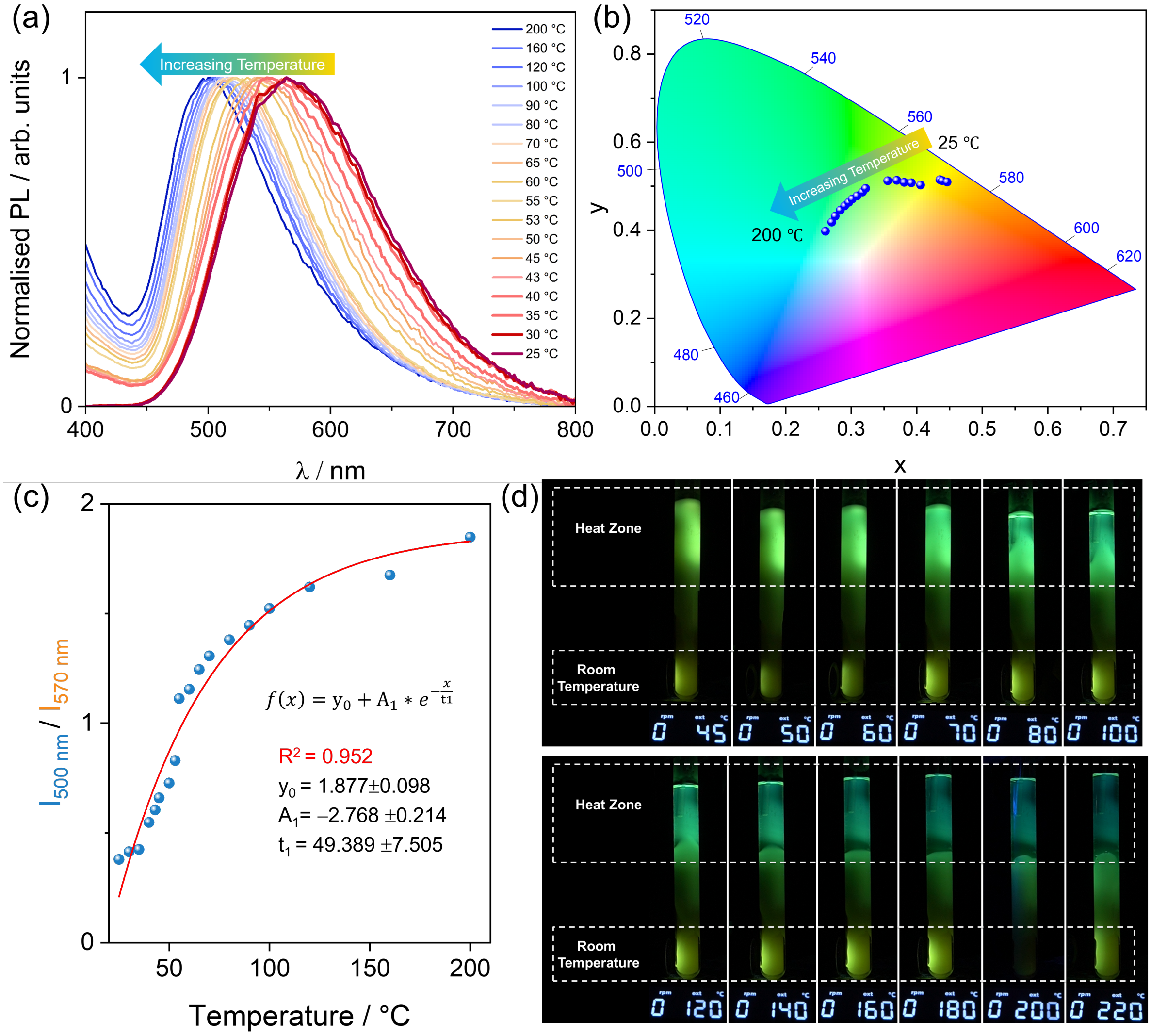

Supplementary Fig. 40. (a) Temperature-dependent emission spectra of Paraffin embedded with 2GCzBPPZ (λ_{exc} = 340 nm); (b) Corresponding CIE plot; (c) Ratiometric plot of I_{500}/I_{570} vs temperature upon increasing the temperature from room temperature to 200 °C; (d) Paraffin with 2GCzBPPZ in test tube (length: 160 mm, diameter: 16 mm) excited with a UV torch (λ_{exc}= 360 nm); The top side (Heat Zone) of the test tube was heated with a heat gun and the temperature was monitored using a Heidolph 509-67910-00 Pt 1000 Temperature Sensor.

For 2GCzBPN, both the fluorescence and phosphorescence spectra are broad and structureless (Supplementary Fig. 41a), indicating that both the S_1 and T_1 states have a CT character. However, the phosphorescence spectrum of 2GCzBPPZ is structured, suggesting that the T_1 state has LE character, while the S_1 state is CT in character. The S_1/T_1 energy levels of 2GCzBPPZ (2.76/2.50 eV) and 2GCzBPN (2.38/2.26 eV) were inferred from the onsets of the prompt fluorescence and phosphorescence spectra, respectively, at 77 K (Supplementary Figs. 41b and 41c). The corresponding rate constants of intersystem crossing (k_{ISC}) for both compounds in mCP films are 1.61×10^7 and 0.45×10^7 s^{-1} for 2GCzBPPZ, and 2GCzBPN, respectively, while the rate constants of RISC (k_{RISC}) for 2GCzBPN reached 4.1×10^5 s^{-1}, a value 20 times faster than in 2GCzBPPZ of 1.93×10^4 s^{-1}, due to the much smaller ΔE_{ST} of 2GCzBPN (0.12 eV) than that of 2GCzBPPZ (0.26 eV).[23]

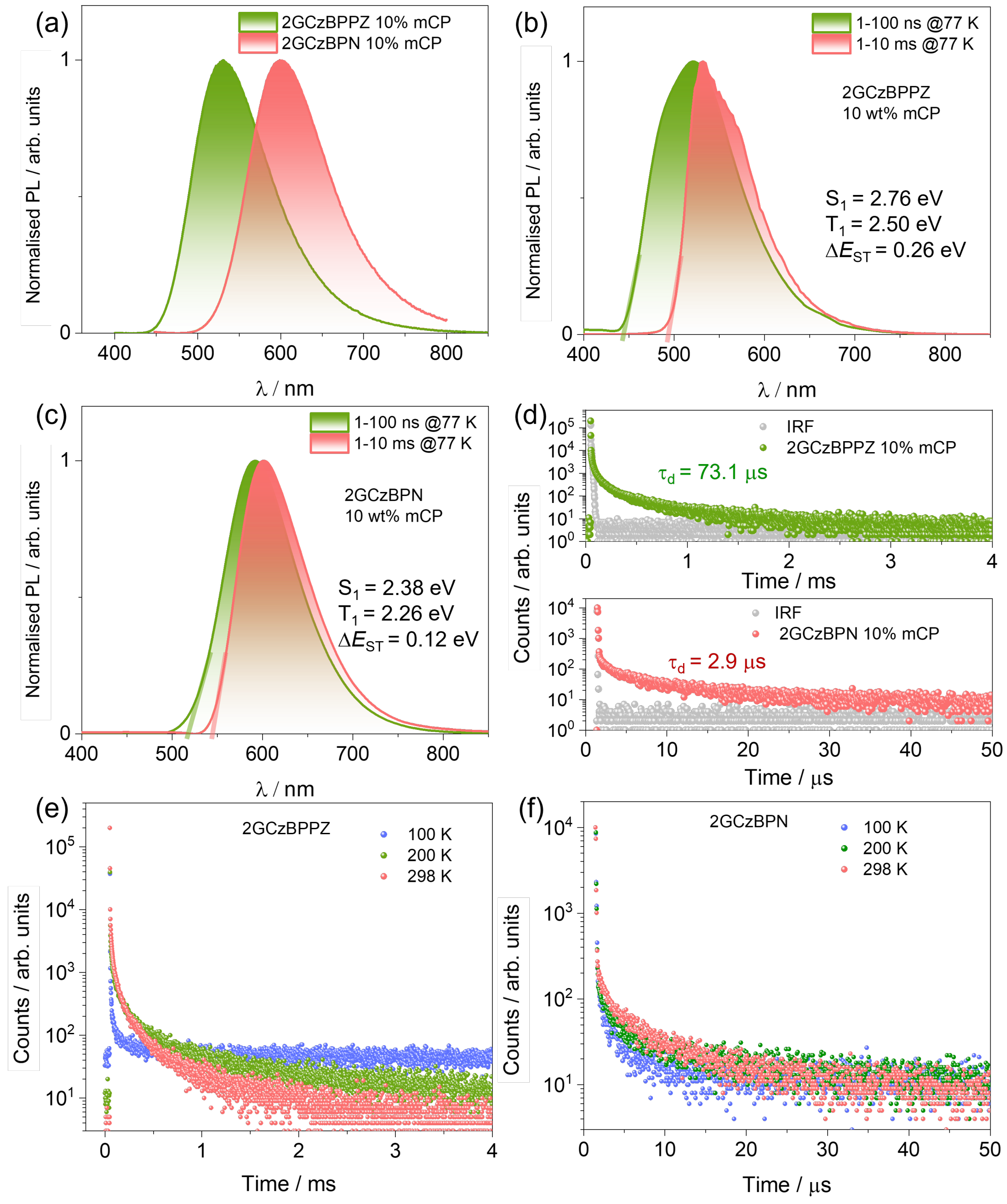

Supplementary Fig. 41. Prompt fluorescence (1-100 ns) and phosphorescence spectra (1-10 ms) in 10 wt% doped in mCP at 77 K of (a) 2GCzBPPZ and (b) 2GCzBPN (λ_{exc} = 345 nm). (c) Steady-state PL spectra of 10 wt% doped films of 2GCzBPPZ and 2GCzBPN in mCP film at room temperature (λ_{exc} =345 nm); (d) PL decay of 10 wt% doped films of 2GCzBPPZ and 2GCzBPN in mCP at 298 K (λ_{exc} =375 nm); Temperature-dependent PL decay of 10 wt% doped films of (e) 2GCzBPPZ and (f) 2GCzBPN in mCP (λ_{exc}= 375 nm).

OLED Characterisation

We followed the guidance from the Osilla site (https://www.ossila.com/pages/oled-testing-guide?_pos=12&_sid=ae5876358&_ss=r) for the OLED Characterisation.

From the three measured parameters, it is possible to calculate the following quantities: Current density(J):

$$J = \frac{i}{\text{device area}}$$

Current efficiency (CE):

$$CE = \frac{Luminescence}{J}$$

Power efficiency (PE):

$$PE = \frac{Luminescence \times \text{device area} \times \pi}{i \times V}$$

The EQE is calculated according to the formula below by assuming that the emission obeys a Lambertian profile,[24-26]

$$\text{EQE} = \frac{\pi L \text{e}}{\text{K}_{\text{m}}\text{hc}J} \frac{\int I(\lambda)\lambda \text{d}\lambda}{\int I(\lambda)V(\lambda)\text{d}\lambda}$$

where e is the electron charge, L is the total luminance, h is the Planck constant, c is the velocity of light and K_m = 683 lm W^{-1} is the maximum luminous efficacy. $I(\lambda)$ is the relative electroluminescence intensity at wavelength λ. $V(\lambda)$ is the normalized photonic spectral response function.

Solution-processed OLEDs, employing 10 wt% emitters doped in mCP films as the emissive layer (EML) were fabricated using the following device stack: ITO (indium tin oxide)/ poly(3,4-ethylenedioxythiophene):poly(styrenesulfonate) (PEDOT:PSS) (40 nm)/ mCP (60%): oxadiazolyl]phenylene (OXD-7) (30%): 10 wt% emitters (20 nm)/ (3,3′-(5′-(3-(pyridin-3-yl)phenyl)-[1,1′:3′,1″-terphenyl]-3,3″-diyl)dipyridine) (TmPyPB) (45 nm)/LiF (1 nm)/Al (Supplementary Fig. 42a and b), where ITO and Al serve as the anode and cathode, respectively, and PEDOT:PSS and TmPyPB are the hole-transporting layer electron-transporting layer, respectively.

As shown in Supplementary Fig. 42b, the OLEDs with **2GCzBPPZ** and **2GCzBPN** exhibit green and orange emission with emission maxima, λ_{EL}, of 552 and 608 nm and corresponding CIE coordinates of (0.39, 0.55) and (0.58, 0.40), respectively, which match the PL emission

(Supplementary Fig. 42c). The devices with 2GCzBPPZ showed lower turn-on voltages (V_{on}) of 3.5 V and high brightness of up to 8000 cd m^{-2} compared to V_{on} of 4.1 V and maximum luminance of 1068 cd m^{-2} for the devices with 2GCzBPN (Supplementary Fig. 42c and Supplementary Table 5). At the same voltages, the current density (*J*) of the 2GCzBPN-based device is lower than that of the 2GCzBPPZ-based device, which we ascribe to the relatively lower electron mobility of 2GCzBPN in the mCP film. The devices with 2GCzBPPZ showed a higher maximum external quantum efficiency (EQE_{max}) of 15.0% at 581 cd m^{-2} with negligible efficiency roll-off at 1000 cd m^{-2} (EQE_{1000} = 14.0%), while the devices with 2GCzBPN showed poorer performance, with EQE_{max} of 5.3% (Supplementary Fig. 42d). Considering the measured Φ_{PL} (Supplementary Table 1) and assuming 25% outcoupling efficiency associated with an isotropic orientation of the transition dipole moment of the emitter,[27,28] the EQE_{max} for devices with 2GCzBPPZ and 2GCzBPN were expected to be 14.3% and 17.8%, respectively. This indicates that the device with 2GCzBPPZ has effectively unity exciton utilization efficiency, however, at this stage it is unclear why the EQE_{max} for 2GCzBPN is so low. We speculate surface plasmons and waveguide and substrate modes are responsible for the light loss (Supplementary Fig. 43). Besides, compared to 2GCzBPPZ, 2GCzBPN with its lower linearity and planarity likely results in a lower light outcoupling efficiency.[29,30]

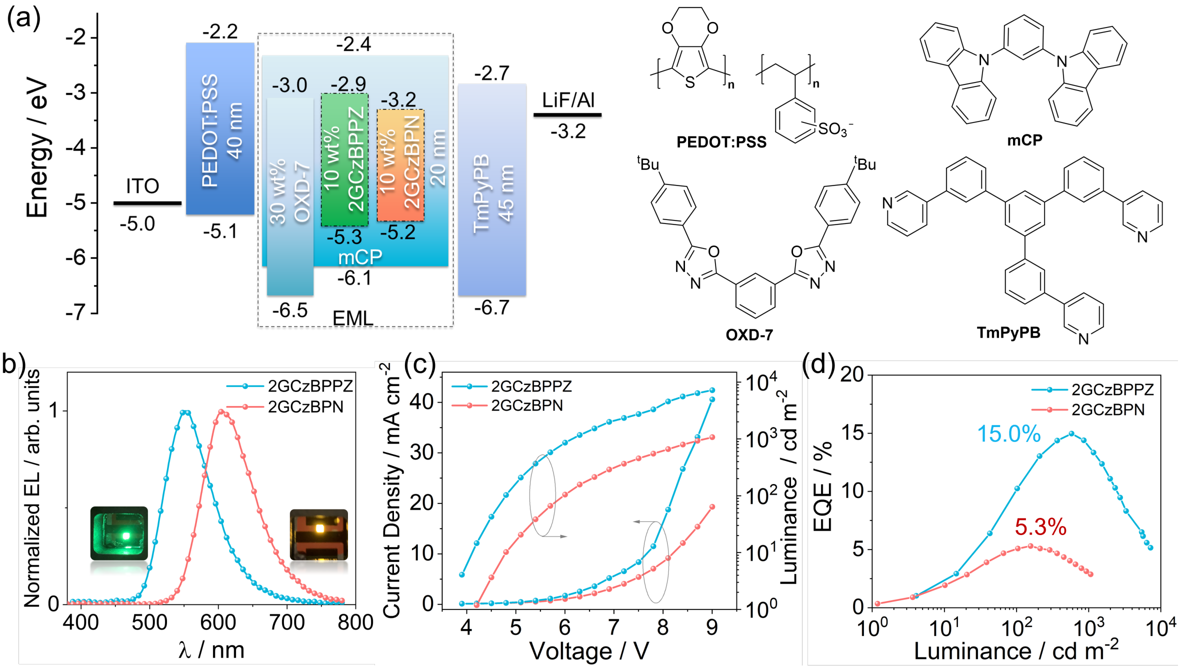

Supplementary Fig. 42. (a) Energy level diagram and structure of materials used in the devices; (b) EL spectra, (c) Current density and luminance versus voltage characteristics, (d) External quantum efficiency versus luminance curves for the devices.

Supplementary Table 5. Electroluminescence data for the devices

Emitter	V_{on} [a] / V	λ_{EL} [b] / nm	L_{max}/ cd m^{-2}	CE / cd A^{-1}	PE_{max} / lm W^{-1}	EQE[c] / %	CIE[d] / x,y
2GCzBPPZ	3.5	552	7207	51.7	28.8	15.0/10.2/14.0	0.39, 0.55
2GCzBPN	4.1	608	1068	10.2	5.1	5.3/5.1/3.0	0.58, 0.40

[a] The turn-on voltage at a brightness ≈ 1 cd m^{-2}. [b] The electroluminescence maximum recorded at 6 V. [c] EQE_{max}/EQE_{100}/ EQE_{1000}. [d] The CIE coordinates recorded at 6 V

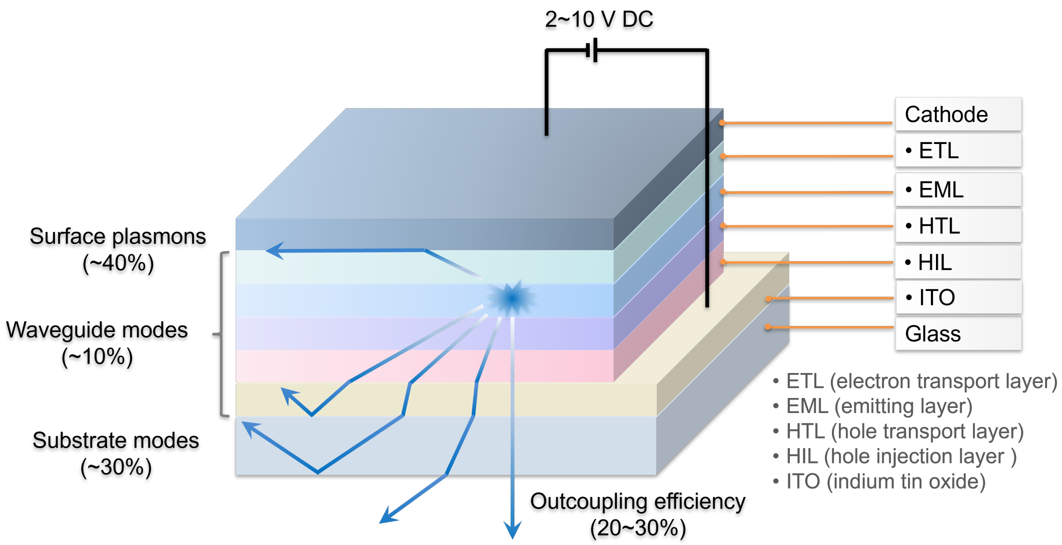

Supplementary Fig. 43. Schematic illustration of the light extraction from OLEDs.

Supplementary References

1. Sun, D., *et al.* Thermally activated delayed fluorescent dendrimers that underpin high-efficiency host-free solution-processed organic light-emitting diodes. *Adv. Mater.* **34**, (2022).
2. Kawata, S., *et al.* Controlling the excited-state energy levels of 9,9′-bifluorenylidene derivatives by twisting their structure to attaining singlet fission character in organic photovoltaics. *J. Mater. Chem. C* **5**, 4909-4914 (2017).
3. Connelly, N. G., Geiger, W. E. Chemical redox agents for organometallic chemistry. *Chem. Rev.* **96**, 877-910 (1996).
4. Dias, F. B., *et al.* The role of local triplet excited states and d-a relative orientation in thermally activated delayed fluorescence: Photophysics and devices. *Adv Sci (Weinh)* **3**, 1600080 (2016).
5. Tsuchiya, Y., *et al.* Exact solution of kinetic analysis for thermally activated delayed fluorescence materials. *J Phys Chem A* **125**, 8074-8089 (2021).
6. Melhuish, W. H. Quantum efficiencies of fluorescence of organic substances: Effect of solvent and concentration of the fluorescent solute1. *J. Phys. Chem.* **65**, 229-235 (1961).
7. Christopherson, C. J., Mayder, D. M., Poisson, J., Paisley, N. R., Tonge, C. M., Hudson, Z. M. 1,8-naphthalimide-based polymers exhibiting deep-red thermally activated delayed fluorescence and their application in ratiometric temperature sensing. *ACS Appl Mater Interfaces* **12**, 20000-20011 (2020).
8. Steinegger, A., Klimant, I., Borisov, S. M. Purely organic dyes with thermally activated delayed fluorescence—a versatile class of indicators for optical temperature sensing. *Adv. Opt. Mater.* **5**, 1700372 (2017).
9. Fister, J. C., Rank, D., Harris, J. M. Delayed fluorescence optical thermometry. *Anal. Chem.* **67**, 4269-4275 (1995).
10. Wang, T., De, J., Wu, S., Gupta, A. K., Zysman-Colman, E. Thermally activated and aggregation-regulated excitonic coupling enable emissive high-lying triplet excitons. *Angew Chem Int Ed Engl* **61**, e202206681 (2022).
11. Si, C., *et al.* Room-temperature multiple phosphorescence from functionalized corannulenes: Temperature sensing and afterglow organic light-emitting diode. *Angew Chem Int Ed Engl* **62**, e202309718 (2023).
12. Lee, D., Bolton, O., Kim, B. C., Youk, J. H., Takayama, S., Kim, J. Room temperature phosphorescence of metal-free organic materials in amorphous polymer matrices. *J. Am. Chem. Soc.* **135**, 6325-6329 (2013).
13. Li, F., *et al.* Color-tunable dual persistent emission via a triplet exciton reservoir for temperature sensing and anti-counterfeiting. *Adv. Opt. Mater.* **10**, 2101773 (2022).
14. Qin, W., Ma, J., Zhou, Y., Hu, Q., Zhou, Y., Liang, G. Simultaneous promotion of efficiency and lifetime of organic phosphorescence for self-referenced temperature sensing. *Chem. Eng.*

J. **400**, (2020).

15. Pais, V. F., Lassaletta, J. M., Fernandez, R., El-Sheshtawy, H. S., Ros, A., Pischel, U. Organic fluorescent thermometers based on borylated arylisoquinoline dyes. *Chemistry* **20**, 7638-7645 (2014).
16. Fang, Q.*, et al.* Thermally populated "bright" states for wide-range and high temperature sensing in air. *Chem. Commun.* **53**, 5702-5705 (2017).
17. Chen, J.*, et al.* A soluble cryogenic thermometer with high sensitivity based on excited-state configuration transformations. *Phys. Chem. Chem. Phys.* **17**, 27658-27664 (2015).
18. Zhu, Q.*, et al.* Reversible thermo-stimulus solid-state fluorescence-colour/on–off switching and uses as sensitive fluorescent thermometers in different temperature ranges. *J. Mater. Chem. C* **4**, 7383-7386 (2016).
19. Wu, Y.*, et al.* Novel ratiometric fluorescent nanothermometers based on fluorophores-labeled short single-stranded DNA. *ACS Appl. Mater. Interfaces* **9**, 11073-11081 (2017).
20. Liu, X., Li, S., Feng, J., Li, Y., Yang, G. A triarylboron-based fluorescent temperature indicator: Sensitive both in solid polymers and in liquid solvents. *Chem. Commun.* **50**, 2778-2780 (2014).
21. Ozawa, A., Shimizu, A., Nishiyabu, R., Kubo, Y. Thermo-responsive white-light emission based on tetraphenylethylene- and rhodamine b-containing boronate nanoparticles. *Chem Commun (Camb)* **51**, 118-121 (2015).
22. Yan, D.*, et al.* Ultrasound-assisted construction of halogen-bonded nanosized cocrystals that exhibit thermosensitive luminescence. *Chem. Eur. J.* **19**, 8213-8219 (2013).
23. Zysman-Colman, E. Molecular designs offer fast exciton conversion. *Nat. Photonics* **14**, 593-594 (2020).
24. Okamoto, S., Tanaka, K., Izumi, Y., Adachi, H., Yamaji, T., Suzuki, T. Simple measurement of quantum efficiency in organic electroluminescent devices. *Jpn. J. Appl. Phys.* **40**, (2001).
25. Forrest, S. R., Bradley, D. D. C., Thompson, M. E. Measuring the efficiency of organic light-emitting devices. *Adv. Mater.* **15**, 1043-1048 (2003).
26. Shen, H.*, et al.* Visible quantum dot light-emitting diodes with simultaneous high brightness and efficiency. *Nat. Photonics* **13**, 192-197 (2019).
27. Tenopala-Carmona, F.*, et al.* Identification of the key parameters for horizontal transition dipole orientation in fluorescent and tadf organic light-emitting diodes. *Adv. Mater.* **33**, 2100677 (2021).
28. Gather, M. C., Reineke, S. Recent advances in light outcoupling from white organic light-emitting diodes. *Journal of Photonics for Energy* **5**, 057607-057607 (2015).
29. Balijapalli, U.*, et al.* Tetrabenzo[a,c]phenazine backbone for highly efficient orange–red thermally activated delayed fluorescence with completely horizontal molecular orientation. *Angew. Chem. Int. Ed.* **60**, 19364-19373 (2021).
30. Woo, J. Y.*, et al.* Advances in solution-processed oleds and their prospects for use in displays.

Adv. Mater. **35**, e2207454 (2023).
