# Supplementary material for: A temperature sensor with a wide spectral range based on a dual-emissive TADF dendrimer system
Source: Nat Commun. 2024 Aug 28;15:7439. doi: 10.1038/s41467-024-51231-x (PMC11358277; doi:10.1038/s41467-024-51231-x)
Supplement: Supplementary file 3 — Description of Additional Supplementary Files [file 41467_2024_51231_MOESM3_ESM.pdf]

Description of Additional Supplementary Files

File Name: Supplementary Data 1
Description: Nuclear coordinates of S0 geometry of 2GCzBPPZ and 2GCzBPN optimized at the PBE0/6-31G(d,p) level in the gas phase.

File Name: Supplementary Movie 1
Description: Dynamic emission color change of 2GCzBPPZ in n-hexane across a temperature range from –70 ℃ to 70 ℃.

File Name: Supplementary Movie 2
Description: Dynamic emission color change of 2GCzBPPZ in paraffin wax upon heating from room temperature.

File Name: Supplementary Movie 3
Description: Spatio-temperature sensor application in paraffin wax.
